# Maternal diabetes-mediated RORA suppression in mice contributes to autism-like offspring through inhibition of aromatase

**DOI:** 10.1038/s42003-022-03005-8

**Published:** 2022-01-13

**Authors:** Hong Yu, Yanbin Niu, Guohua Jia, Yujie Liang, Baolin Chen, Ruoyu Sun, Min Wang, Saijun Huang, Jiaying Zeng, Jianpin Lu, Ling Li, Xiaoling Guo, Paul Yao

**Affiliations:** 1 Department of Pediatrics, Foshan Maternity and Child Health Care Hospital, Foshan, 528041 P. R. China; 2 Teachers College, Columbia University, New York, NY 10027 USA; 3 Hainan Women and Children’s Medical Center, Haikou, 570206 P. R. China; 4 Department of Child Psychiatry, Kangning Hospital of Shenzhen, Shenzhen Mental Health Center, Shenzhen, 518020 P. R. China

**Keywords:** Molecular neuroscience, Autism spectrum disorders

## Abstract

Retinoic acid-related orphan receptor alpha (RORA) suppression is associated with autism spectrum disorder (ASD) development, although the mechanism remains unclear. In this study, we aim to investigate the potential effect and mechanisms of RORA suppression on autism-like behavior (ALB) through maternal diabetes-mediated mouse model. Our in vitro study in human neural progenitor cells shows that transient hyperglycemia induces persistent RORA suppression through oxidative stress-mediated epigenetic modifications and subsequent dissociation of octamer-binding transcription factor 3/4 from the *RORA* promoter, subsequently suppressing the expression of aromatase and superoxide dismutase 2. The in vivo mouse study shows that prenatal RORA deficiency in neuron-specific *RORA* null mice mimics maternal diabetes-mediated ALB; postnatal RORA expression in the amygdala ameliorates, while postnatal *RORA* knockdown mimics, maternal diabetes-mediated ALB in offspring. In addition, *RORA* mRNA levels in peripheral blood mononuclear cells decrease to 34.2% in ASD patients (*n* = 121) compared to the typically developing group (*n* = 118), and the related Receiver Operating Characteristic curve shows good sensitivity and specificity with a calculated 84.1% of Area Under the Curve for ASD diagnosis. We conclude that maternal diabetes contributes to ALB in offspring through suppression of RORA and aromatase, RORA expression in PBMC could be a potential marker for ASD screening.

## Abstract (web summary)

Hong Yu, Yanbin Niu, Guohua Jia et al. integrate in vitro, in vivo, and human experiments to examine a link between *RORA* expression on autism-like behavior. Their results suggest that maternal diabetes may contribute to autism-like behavior via *RORA* suppression.

## Introduction

During the past few decades, the prevalence of autism spectrum disorders (ASD) has significantly increased to 1:54^1^, with a male:female ratio of >4:1^2,3^. A variety of potential risk factors, including genetic/epigenetics, sex, and environmental exposure, have been reported to be associated with ASD development^4,5^. We have previously reported that factors such as maternal diabetes and elevated prenatal exposure to hormones, including progestin and androgen^6–8^, contribute to ASD development through epigenetic modifications and subsequent gene suppression and oxidative stress in offspring^9,10^, while the detailed mechanism still needs to be further investigated^11^.

Retinoic acid-related orphan receptor alpha (RORA) belongs to the nuclear receptor superfamily and is involved with a variety of pathophysiological processes^12^, playing a critical role in development, immunity, and cellular metabolism^13^. Epidemiological studies have shown that RORA is associated with ASD development^14^, and it has also been reported that RORA expression is reduced in ASD patients and contributes to sex bias and ASD development by regulation of target gene cytochrome P450, family 19 (*CYP19A1*, aromatase). Additionally, findings have indicated that RORA suppression may be due to risk factor-mediated epigenetic modifications on the *RORA* promoter, while the detailed mechanism is still unknown^15–17^.

Expression of aromatase (encoded by *CYP19* gene), the rate-limiting enzyme that converts androgens to estradiol (E2)^18^, has been reported to be reduced in ASD patients^19^. This can be explained by the hypothesis that aromatase is transcriptionally regulated by RORA in the brain^20^. Additionally, it has been reported that E2 and estrogen receptor (ER) regulates superoxide dismutase 2 (SOD2) expression through estrogen response element (ERE) on the SOD2 promoter^21^ and that maternal diabetes-mediated SOD2 suppression contributes to autistic offspring^22^. We hypothesize that RORA suppression may contribute to ASD development through aromatase/E2-mediated SOD2 suppression and subsequent reactive oxygen species (ROS) generation^23^.

In this paper, we aim to investigate the potential effect and mechanism of RORA on ASD development through three distinct studies: an in vitro study looking at human progenitor cells, an in vivo study using a mouse model, and a human study comparing *RORA* mRNA levels in autistic and control children. Our in vitro study through human neural progenitor cells showed that RORA expression was suppressed through hyperglycemia-induced epigenetic modification on the *RORA* promoter. CYP19A1 was regulated by RORA through RORE (retinoic acid-related orphan receptor elements) on the *CYP19A1* promoter. The in vivo mouse study showed that prenatal RORA deficiency mimicked maternal diabetes-mediated autism-like behavior (ALB); postnatal expression of RORA in amygdala ameliorated, while postnatal knockdown of RORA mimicked, maternal diabetes-mediated ALB. In addition, the human study showed that *RORA* mRNA levels in peripheral blood mononuclear cells (PBMC) decreased to 34.2% in the ASD group compared to the typically developing (TD) group. We conclude that maternal diabetes contributes to ALB in offspring through the suppression of RORA and aromatase.

## Results

### In vitro cell study: transient hyperglycemia causes persistent suppression of RORA and its target genes during subsequent normoglycemia

**Fig. 1:**
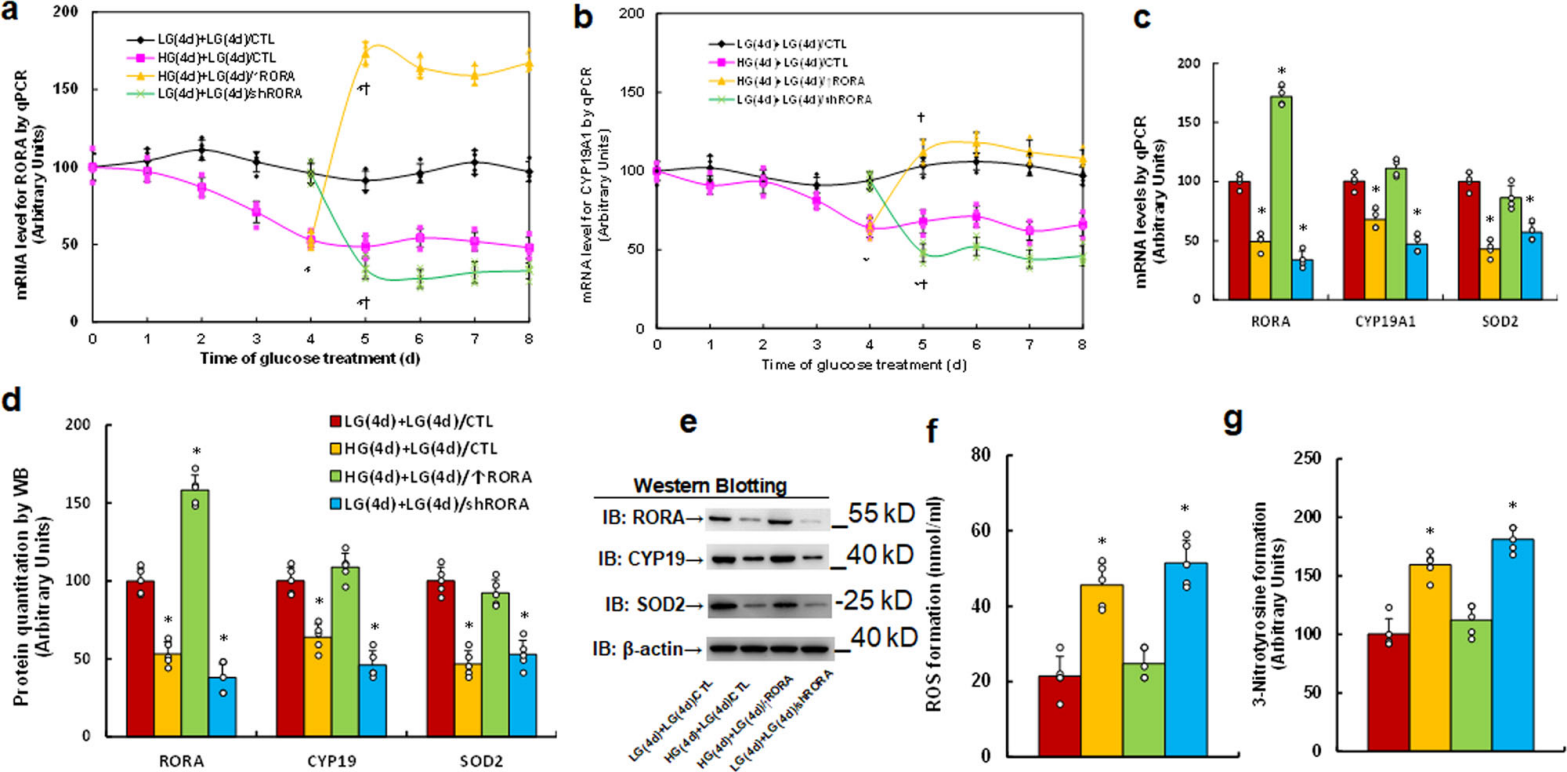
Transient hyperglycemia causes persistent suppression of RORA and its target genes during subsequent normoglycemia. Human ACS-5003 neurons were treated with either 5 mM low glucose (LG) or 25 mM high glucose (HG) for 4 days. The cells were then infected by empty (CTL), RORA overexpression (↑RORA), or RORA knockdown (shRORA) lentivirus for one day before treatment with LG for another 4 days in the presence of 1% serum; the cells were then harvested for further analysis. **a**, **b** Cells were harvested at different time points for analysis of mRNA levels. **a** RORA levels; **b** CYP19A1 levels; *n* = 4, *, *P* < 0.0001, vs. day 0 group; ¶, *P* < 0.0001, vs. day 4 group. **c**–**g** Cells were harvested on day 8 for biomedical analysis. **c** mRNA levels, *n* = 4. **d** Quantitation of protein levels, *n* = 5. **e** Representative western blotting pictures for (**d**). **f** ROS formation, *n* = 5. **g** 3-nitrotyrosine formation, *n* = 5. *, *P* < 0.0001, vs. LG(4d)+LG(4d)/CTL group. Data were expressed as mean ± SEM.

We evaluated the potential effect of hyperglycemia on the expression of RORA and its target genes *CYP19A1* and *SOD2*. Human ACS-5003 neurons were treated by 25 mM high glucose (HG) for 4 days followed by subsequent 5 mM low glucose for another 4 days before biomedical analysis. Gene expression with time-dependent response curves showed that 4-day HG treatment significantly suppressed the gene expression of *RORA* (see Fig. 1a), *CYP19A1* (see Fig. 1b), and *SOD2* (see Fig. 1c), and gene expression remained low during subsequent 4-day normoglycemia. Infection of RORA expression lentivirus on day 5 (HG(4d)+LG(4d)/↑RORA) significantly increased *RORA* expression and completely restored hyperglycemia-induced suppression of *CYP19A1* and *SOD2*, while infection of RORA knockdown lentivirus on day 5 (LG(4d)+LG(4d)/shRORA) significantly decreased expression of *RORA*, *CYP19A1*, and *SOD2* compared to the control (LG(4d)+LG(4d)/CTL) group, and mRNA levels of *RORA*, *CYP19A1*, and *SOD2* on day 8 are presented in Fig. 1c. We then evaluated the protein levels for RORA, CYP19, and SOD2, and an expression pattern similar to that of the mRNA was observed (see Fig. 1d, e, Supplementary Fig. 1a). Finally, we evaluated the potential effect of hyperglycemia and RORA expression on oxidative stress, and the results showed that transient hyperglycemia HG(4d)+LG(4d)/CTL treatment significantly increased ROS formation (see Fig. 1f) and 3-nitrotyrosine formation (see Fig. 1g) compared to the control (LG(4d)+LG(4d)/CTL) group, and RORA expression (HG(4d)+LG(4d)/↑RORA completely reversed, while RORA knockdown (LG(4d)+LG(4d)/shRORA mimicked the transient hyperglycemia-mediated effect. Our results suggest that transient hyperglycemia triggers persistent RORA suppression during subsequent normoglycemia and that the expression of CYP19A1 and SOD2 is regulated by RORA.

### In vitro cell study: hyperglycemia induces RORA suppression through epigenetic modification and the subsequent dissociation of Oct3/4 from the *RORA* promoter

**Fig. 2:**
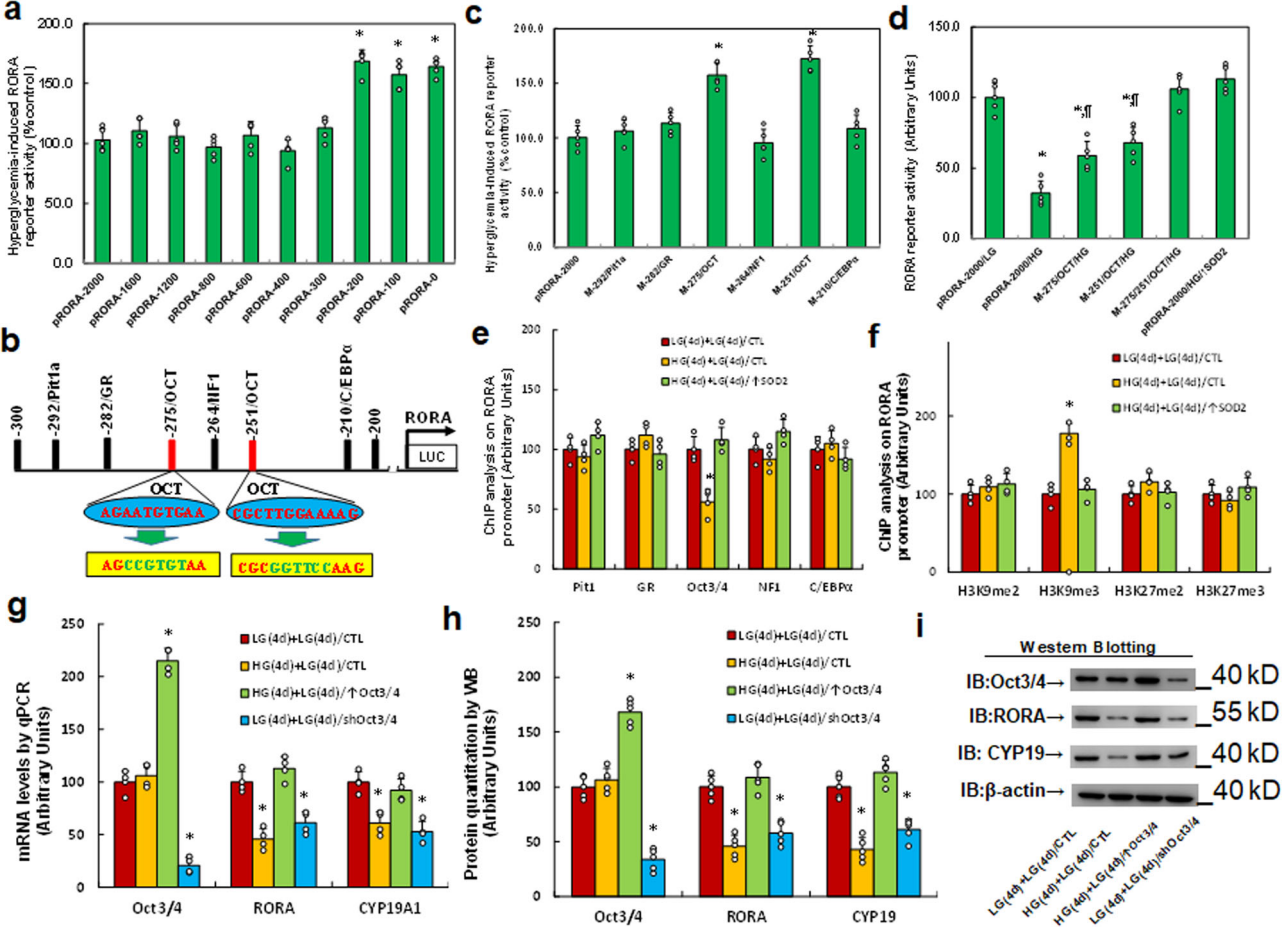
Hyperglycemia induces RORA suppression through epigenetic modification and the subsequent dissociation of Oct3/4 from the RORA promoter. **a** The conditional immortalized ACS-5003 neurons were transiently transfected with either RORA full length (pRORA-2000) or deletion reporter plasmids. After 24 h, the cells were treated with either 5 mM low glucose (LG) or 25 mM high glucose (HG) for 3 days and the relative RORA reporter activities were calculated *n* = 5. *, *P* < 0.05, vs. pRORA-2000 group. **b** The schematic picture for the potential transcriptional binding motif in the range of −300 to 200 (from transcription start site) on the *RORA* promoter with two potential Oct binding sites marked in red as well as related mutation sites marked in green. **c** The cells were transiently transfected by either a wild-type *RORA* reporter construct (pRORA-2000) or single point mutation at the site shown in (**b**), and then treated with either LG or HG for 3 days, and the RORA reporter activities were calculated *n* = 5. *, *P* < 0.05, vs. pRORA-2000 group. **d** The cells were transiently transfected by RORA full length (pRORA-2000), single mutant, or double mutations as indicated, or infected by SOD2 lentivirus (↑SOD2), and then treated with either LG or HG for 3 days; the RORA reporter activities were then calculated, *n* = 5. *, *P* < 0.0001, vs. pRORA-2000/LG group; ¶, *P* < 0.001, vs. pRORA-2000/HG group. **e**, **f** Cells were treated by either 4-day LG plus 4-day LG (LG(4d)+LG(4d)), or 4-day HG plus 4-day LG (HG(4d)+LG(4d)), or infected on day 4 by SOD2 lentivirus (HG(4d)+LG(4d)/↑SOD2); the cells were then used for ChIP analysis: **e** ChIP analysis by potential transcription factors on the *RORA* promoter, *n* = 4; **f** ChIP analysis by potential histone methylation, *n* = 4. *, *P* < 0.0001, vs. LG(4d)+LG(4d)/CTL group. **g**–**i** Cells were treated by either LG(4d)+LG(4d)/CTL or HG(4d)+LG(4d)/CTL, or the cells were infected on day 4 by either Oct3/4 expression lentivirus (HG(4d)+LG(4d)/↑Oct3/4) or Oct3/4 knockdown lentivirus (LG(4d)+LG(4d)/shOct3/4); the cells were then harvested for biomedical analysis: **g** mRNA analysis, *n* = 4. **h** Protein quantitation, *n* = 5. **i** Representative western blotting pictures for (**h**). *, *P* < 0.0001, vs. LG(4d)+LG(4d)/CTL group. Data were expressed as mean ± SEM.

We investigated the potential molecular mechanism for transient hyperglycemia-mediated persistent RORA suppression. The *RORA* promoter was used to generate a series of progressive 5′-promoter deletion constructs for subsequent transfection into conditionally immortalized neurons for the analysis of *RORA* reporter activity with the 24-h treatment of either 5 mM LG or 25 mM HG, and the relative reporter activity was calculated by the ratio of reporter activity at HG divided by activity at low glucose (% control). Our results showed that hyperglycemia-mediated RORA reporter suppression occurred in the −2000, −1600, −1200, −800, −600, −400, and −300 deletion constructs (numbered according to Ensembl gene ID: RORA-201 ENST00000261523.9; transcription start site (TSS) was marked as 0), while the suppression completely disappeared in the −200 and subsequent deletion reporter constructs, suggesting that hyperglycemia-responsive transcriptional element is located in the range of −300 to −200 on the *RORA* promoter (see Fig. 2a). We then searched the transcription factor database and identified a variety of potential binding motifs, including one of each for Pit1a, GR, NF1, and C/EBPα, and two for OCT (marked in red), which were located in the range of −300 to −200 on the *RORA* promoter (see Fig. 2b). The related mutations for each of the identified binding motifs in the *RORA* full length (pRORA-2000) reporter construct were generated. The reporter assay showed that hyperglycemia-induced reporter suppression disappeared in two of the OCT mutation constructs (located at −276 and −251, respectively, marked in green, see Fig. 2b), suggesting that hyperglycemia mediates RORA suppression through the OCT binding motif on the *RORA* promoter (see Fig. 2c). The related single or double mutations on the two OCT binding motifs (located at −276 and −251) were generated in the *RORA* full length construct, and the reporter assay showed that OCT single mutants (M-276/OCT and M-251/OCT) significantly increased *RORA* reporter activity in the HG treatment group compared to the wild type full length with LG treatment (pRORA-2000/LG), while OCT double mutants (M-276/-251/OCT/HG) completely reversed HG treatment (pRORA-2000/HG)-mediated RORA suppression (see Fig. 2d), indicating that hyperglycemia induces RORA suppression through the OCT binding motif on the *RORA* promoter. We then measured DNA methylation on the *RORA* promoter and found that there was no significant difference for different treatments (see Supplementary Fig. 2). We also conducted ChIP analysis on the *RORA* promoter using antibodies for Pit1, GR, Oct3/4, NF1, and C/EBPα and found that the binding ability of Oct3/4 on the RORA promoter decreased to 56% in the HG(4d)+LG(4d)/CTL group compared to the LG(4d)+LG(4d)/CTL group. This effect was completely reversed by SOD2 expression (HG(4d)+LG(4d)/↑SOD2), while other transcription factors showed no significant difference (see Fig. 2e), suggesting that Oct3/4 dissociation from the *RORA* promoter is responsible for hyperglycemia-mediated RORA suppression. We further evaluated the possible epigenetic changes on the *RORA* promoter, and the results showed that there was no effect on histone H4 methylation (see Supplementary Fig. 3a) or histone acetylation on the *RORA* promoter (see Supplementary Fig. 3b). We then evaluated the effect of hyperglycemia on histone H3 methylation and found that hyperglycemia treatment increased H3K9me3 methylation to 178% in HG(4d)+LG(4d)/CTL treatment compared to the control (LG(4d)+LG(4d)/CTL) group, while it showed no effect on the methylation of H3K9me2, H3K27me2, and H3K27me3. Furthermore, the expression of SOD2 (HG(4d)+LG(4d)/↑SOD2) completely reversed this effect (see Fig. 2f). Finally, we evaluated the potential effect of Oct3/4 on RORA expression. Oct3/4 expression was successfully manipulated by either Oct3/4 overexpression or knockdown lentivirus, and we found that Oct3/4 expression after HG treatment (HG(4d)+LG(4d)/↑Oct3/4) completely reversed, while Oct3/4 knockdown after LG treatment (LG(4d)+LG(4d)/shOct3/4) mimicked, hyperglycemia treatment (HG(4d)+LG(4d)/CTL)-mediated RORA suppression compared to the control (LG(4d)+LG(4d)/CTL) group (see Fig. 2g–i, Supplementary Fig. 1b). We conclude that hyperglycemia induces RORA suppression through epigenetic modification and the subsequent dissociation of Oct3/4 from the *RORA* promoter.

### In vitro cell study: hyperglycemia induces CYP19A1 suppression through RORA dissociation from the RORE element on the *CYP19A1* promoter

**Fig. 3:**
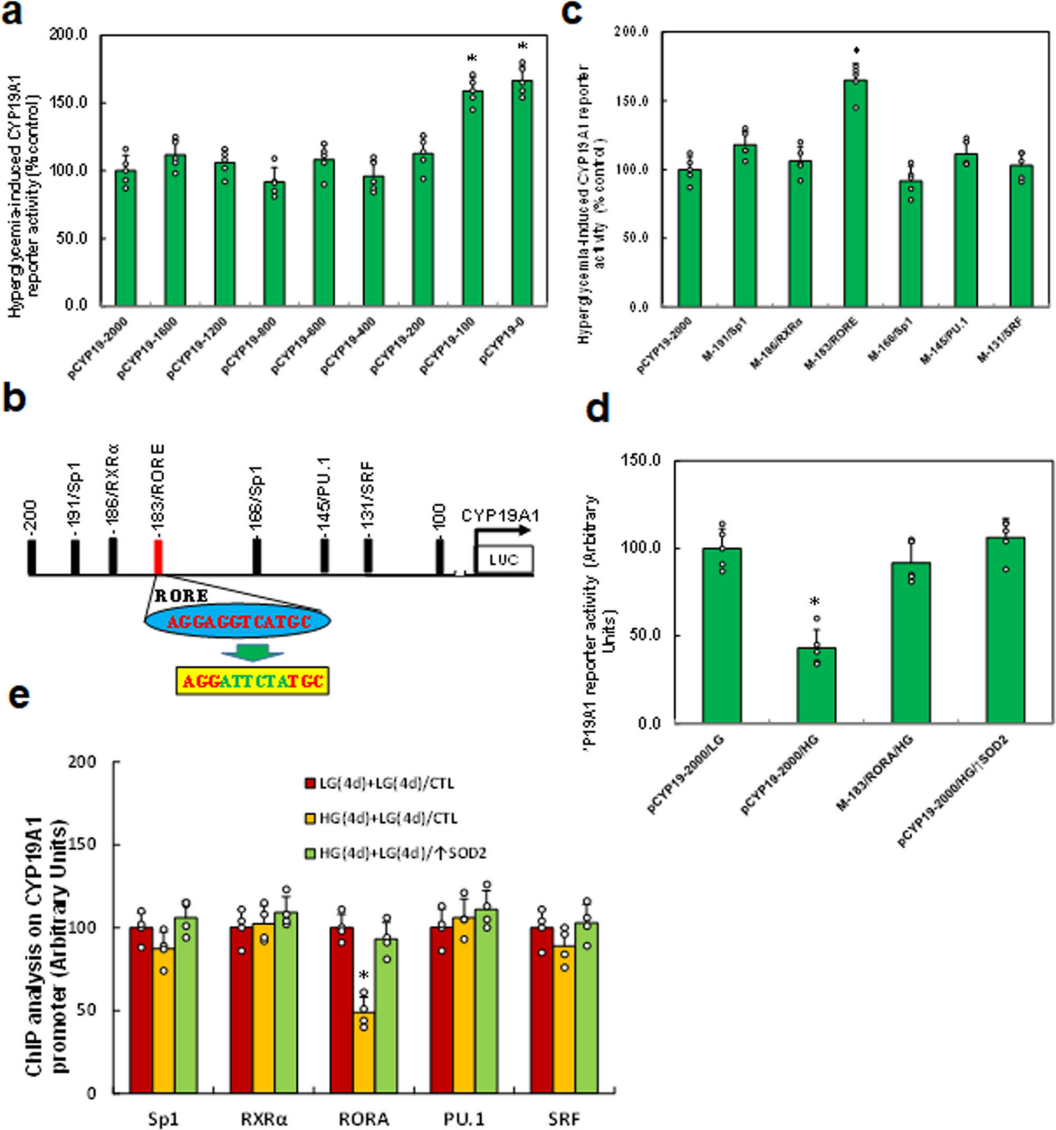
Hyperglycemia induces CYP19A1 suppression through RORA dissociation from the RORE response element on the CYP19A1 promoter. **a** The conditional immortalized ACS-5003 neurons were transiently transfected with either CYP19A1 full length (pCYP19-2000) or deletion reporter plasmids. After 24 h, the cells were treated with either 5 mM low glucose (LG) or 25 mM high glucose (HG) for 3 days and the relative CYP19A1 reporter activities were calculated *n* = 5. *, *P* < 0.0001, vs. pCYP19-2000 group. **b** The schematic picture for the potential transcriptional binding motif in the range of −200 to 100 (from transcription start site) on the CYP19 promoter with one potential RORA response element (RORE) marked in red as well as related mutation site marked in green. **c** The cells were transiently transfected by either a wild-type CYP19 reporter construct (pCYP19-2000) or single point mutation at the site shown in (**b**), and then treated with either LG or HG for 3 days, and the CYP19A1 reporter activities were calculated *n* = 5. *, *P* < 0.0001, vs. pCYP19-2000 group. **d** The cells were transiently transfected by CYP19A1 full length (pCYP19-2000), single mutant, or infected by SOD2 lentivirus (↑SOD2), and then treated with either LG or HG for 3 days; the CYP19A1 reporter activities were then calculated, *n* = 5. *, *P* < 0.0001, vs. pCYP19A1-2000/LG group. **e** Cells were treated with either 4-day LG plus 4-day LG (LG(4d)+LG(4d)), or 4-day HG plus 4-day LG (HG(4d)+LG(4d)), or infected on day 4 by SOD2 lentivirus (HG(4d)+LG(4d)/↑SOD2); the cells were then used for ChIP analysis by potential transcription factors on the *CYP19A1* promoter, *n* = 4. *, *P* < 0.0001, vs. LG(4d)+LG(4d)/CTL group. Data were expressed as mean ± SEM.

We evaluated the potential molecular mechanism for hyperglycemia-mediated CYP19A1 suppression. A series of progressive 5′-promoter deletion constructs for the *CYP19A1* promoter were generated, and they were transfected into immortalized neurons for reporter activity assay in the presence of either 5 mM LG or 25 mM HG for 24 h. The results showed that hyperglycemia-mediated *CYP19A1* reporter suppression occurred in the −2000, −1600, −1200, −800, −600, −400, and −200 deletion constructs (numbered according to Ensembl gene ID: CYP19A1-201 ENST00000396402.6; TSS was marked as 0), while the suppression disappeared in the −100 deletion construct, indicating that hyperglycemia-responsive element was located in the range of −200 to −100 on the *CYP19A1* promoter (see Fig. 3a). All the potential transcriptional binding motifs in this area were identified from the transcription factor database as the following: two for Sp1, and one of each for RXRα, PU.1, SRF, and RORE (marked in red, see Fig. 3b). The related motif deletion constructs were then generated from *CYP19A1* full-length reporter construct (pCYP19-2000) for reporter assay, and the results showed that hyperglycemia-induced reporter suppression disappeared in the RORE deletion construct (M-183/RORE) (see Fig. 3c). Furthermore, the single mutant for RORE elements (M-183/RORE, see Fig. 3b, marked in green) was generated from the *CYP19A1* full-length promoter for reporter assay. The results showed that RORE mutation (M-183/RORE/HG) completely reversed hyperglycemia-mediated CYP19A1 suppression, indicating that hyperglycemia suppresses CYP19A1 through the RORE element (located at −183) on the *CYP19A1* promoter (see Fig. 3d). We then evaluated the binding abilities by ChIP analysis on the *CYP19A1* promoter using antibodies for the following transcription factors: Sp1, RXRα, RORA, PU.1, and SRF and found that the binding ability of RORA on the *CYP19A1* promoter decreased to 49% as a result of hyperglycemia treatment (HG(4d)+LG(4d)/CTL) compared to control (LG(4d)+LG(4d)/CTL) treatment. This effect was reversed by SOD2 expression (HG(4d)+LG(4d)/↑SOD2), while other transcription factors showed no effect (see Fig. 3e). We conclude that hyperglycemia induces CYP19A1 suppression through RORA dissociation from the RORE element on the *CYP19A1* promoter.

### In vivo mouse study: prenatal RORA deficiency mimics maternal diabetes-mediated oxidative stress

**Fig. 4:**
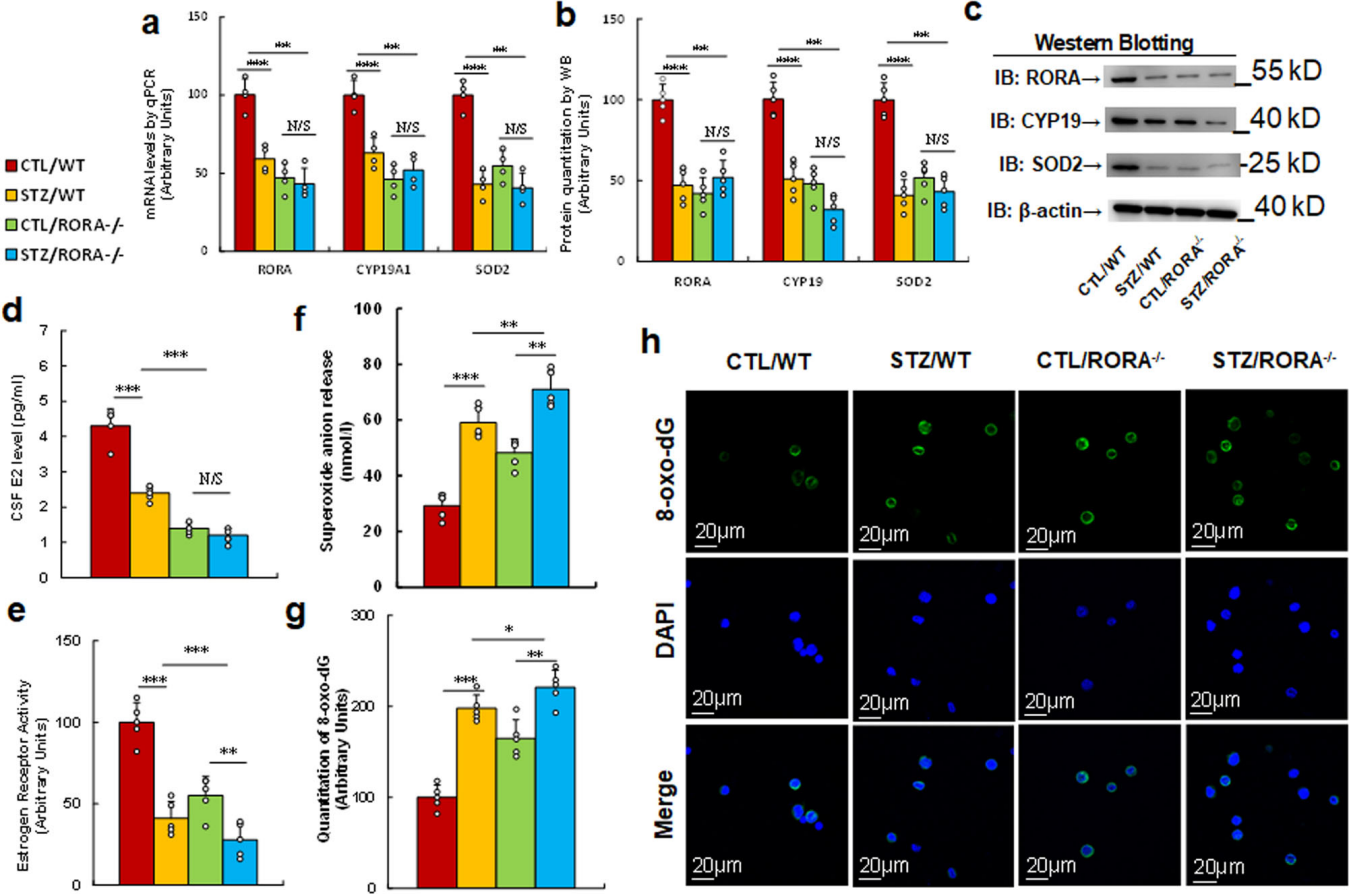
Prenatal RORA deficiency mimics maternal diabetes-mediated oxidative stress. The RORA wild type (WT) or RORA null (RORA^−/−^) backgrounds were used to generate either control (CTL) or STZ-induced diabetic (STZ) pregnant dams, and the amygdala neurons and/or tissues from subsequent male offspring were isolated for further analysis. **a**–**d** The amygdala tissues were isolated from 7- to 8-week-old male offspring for analysis. **a** The mRNA levels by qPCR, *n* = 4. **b** The quantitation of protein levels, *n* = 5. **c** The representative pictures for western blotting for (**b**). **d** CSF E2 levels, *n* = 9. **e** Estrogen receptor activity, *n* = 9. **f** In vivo superoxide anion release, *n* = 5. **g**, **h** The amygdala neurons were isolated on an embryonic day (E18) from the above treatment for immunostaining. **g** Quantitation of 8-oxox-dG staining, *n* = 5. **h** Representative pictures for 8-oxo-dG staining (green) and DAPI staining for nuclei (blue). ***, *P* < 0.0001; **, *P* < 0.001; *, *P* < 0.01; N/S, no significance. Data were expressed as mean ± SEM.

The *RORA* wild type (WT) and *RORA* null (*RORA*^−/−^) mice were crossbred with Otp^Cre^ mice (#030557) to generate amygdala-specific RORA^−/−^ null mice (Otp^Cre^-*RORA*^fl/fl^). Different parts of the amygdala, including the medial, lateral, and basolateral sections, were isolated for analysis of *RORA* mRNA by qPCR, and our results showed that the amygdala-specific *RORA*^−/−^ null mouse (Otp^Cre^-*RORA*^fl/fl^) showed significantly decreased RORA expression not only in the medial amygdala but also in the lateral and basolateral amygdala, compared to the WT group (see Supplementary Fig. 4). We then evaluated the potential effect of prenatal RORA deficiency on maternal diabetes-mediated gene expression and oxidative stress. The *RORA* wild type (WT) or *RORA* null (RORA^−/−^) backgrounds were used to generate either control (CTL) or STZ-induced diabetic (STZ) pregnant dams, and the amygdala neurons or tissues from subsequent male offspring were isolated for analysis. We first evaluated gene expression in the amygdala, including mRNA (see Fig. 4a) and protein levels (see Fig. 4b, c, Supplementary Fig. 1c). The results showed that maternal diabetes (STZ/WT) significantly decreased the expression of RORA, CYP19A1, and SOD2 compared to the control (CTL/WT) group, and prenatal RORA deficiency mimicked the effect of maternal diabetes. We also evaluated mRNA expression in the hypothalamus (see Supplementary Fig. 5a) and hippocampus (see Supplementary Fig. 5b). The results showed that the expression of RORA and CYP19A1 significantly decreased in both maternal diabetes (STZ/WT) and prenatal RORA deficiency treatments (including both CTL/RORA^−/−^ and STZ/RORA^−/−^ groups) compared to the control (CTL/WT) group, while SOD2 expression in the hippocampus showed no changes. Moreover, in the hypothalamus, *SOD2* mRNA levels did not significantly change in maternal diabetes (STZ/WT) group, while they were significantly decreased in the prenatal *RORA* deficiency (*RORA*^−/−^) group compared to the control (CTL/WT) group. In addition, we also evaluated the gene expression of *RORA* (see Supplementary Fig. 6a) and *SOD2* (see Supplementary Fig. 6b) in several other brain regions, including the cerebral cortex, ventral striatum, and cerebellum, and the results showed that there was no significant difference in mRNA levels as results of these treatments. We then evaluated the potential effect of RORA on estrogen and ERs. The results showed that maternal diabetes (CTL/WT) significantly decreased CSF estradiol (E2) levels to 56%, and prenatal *RORA* deficiency (*RORA*^−/−^) treatment further decreased CSF E2 levels to around 33%, compared to the control (CTL/WT) group (see Fig. 4d). In addition, maternal diabetes (CTL/WT) significantly decreased ER activity to 41%, and prenatal *RORA* deficiency, including the deficiency displayed in the CTL/RORA^−/−^ and STZ/RORA^−/−^ groups, decreased ER activity to 55 and 28%, respectively, compared to the control (CTL/WT) group (see Fig. 4e). Finally, we evaluated the effect of oxidative stress. The results showed that maternal diabetes (CTL/WT) significantly increased superoxide anion release to 203% compared to the control (CTL/WT) group, and prenatal *RORA* deficiency (*RORA*^−/−^) treatment further potentiated this effect (see Fig. 4f). In addition, maternal diabetes (CTL/WT) significantly increased 8-oxo-dG formation to 198% compared to the control (CTL/WT) group, and prenatal *RORA* deficiency (*RORA*^−/−^) treatment mimicked the effect of maternal diabetes (see Fig. 4g, h). We conclude that prenatal RORA deficiency mimics maternal diabetes-mediated oxidative stress.

### In vivo mouse study: prenatal RORA deficiency partly mimics maternal diabetes-mediated ALB, while it has little effect on anxiety-like behavior in offspring

**Fig. 5:**
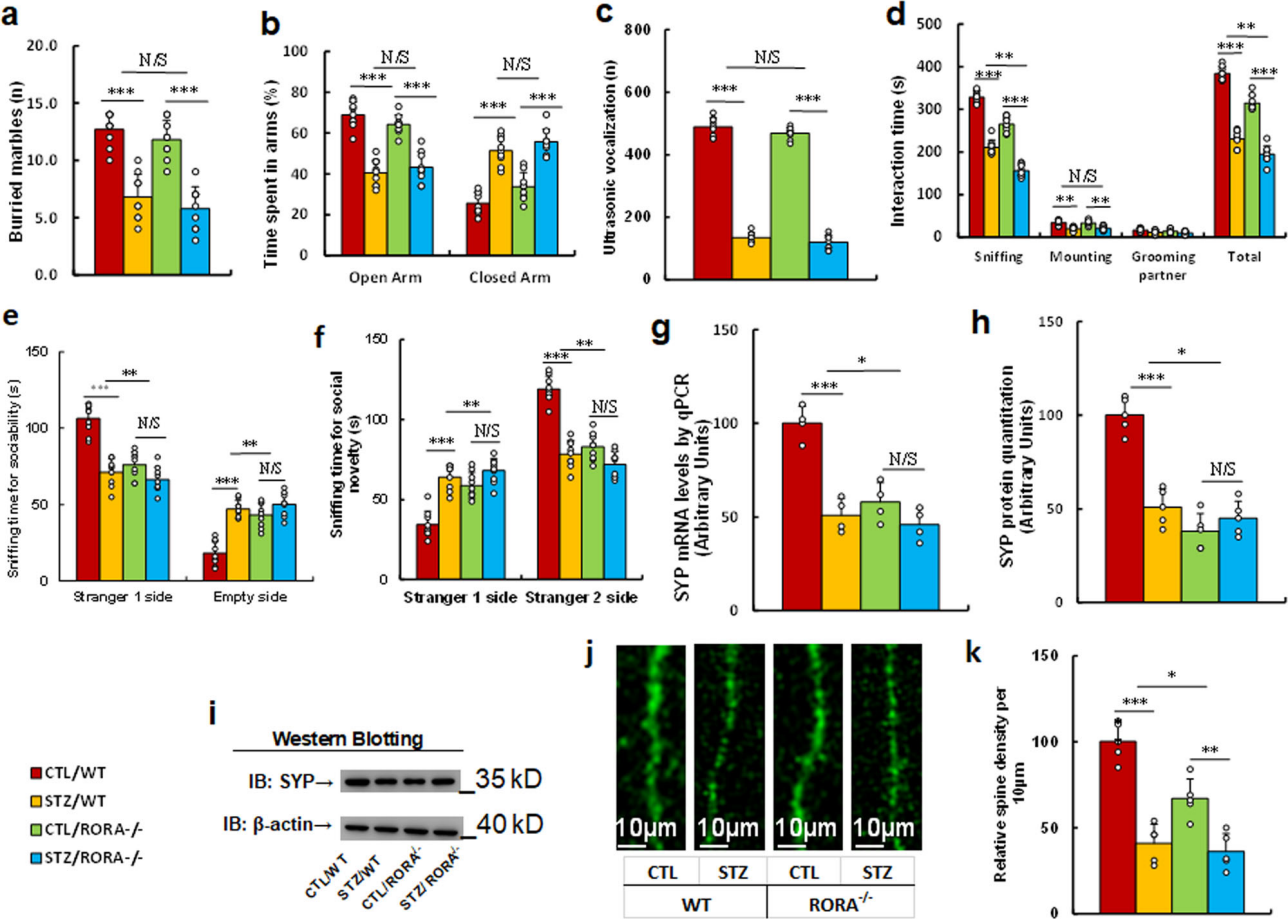
Prenatal RORA deficiency potentiates maternal diabetes-mediated autism-like behavior, while it has little effect on anxiety-like behavior in offspring. The RORA wild type (WT) or RORA null (RORA^−/^^−^) background were used to generate either control (CTL) or STZ-induced diabetic (STZ) pregnant dams, and the subsequent 7- to 8-week-old male offspring were used for animal behavior analysis. **a** Marble-burying tests (MBT), *n* = 9. **b** Time spent in Open Arm and Closed Arm in the EPM test, *n* = 9. **c** Ultrasonic vocalization, *n* = 9. **d** Social interaction (SI) test, the time spent in following, mounting, grooming, and sniffing any body parts of the other mouse, and in total was calculated, *n* = 9. **e**, **f** Three-chambered social tests, *n* = 9. **e** Time spent in the chamber for sociability. **f** Time spent in the chamber for social novelty. **g** SYP mRNA level by qPCR, *n* = 4. **h** SYP protein quantitation, *n* = 5. **i** The representative pictures for western blotting for (**h**). **j** Representative images of dendritic spines in the amygdala. **k** Quantitative data of spine density, *n* = 5. ***, *P* < 0.0001; **, *P* < 0.001; *, *P* < 0.01; N/S no significance. Data were expressed as mean ± SEM.

We evaluated the potential effect of prenatal RORA deficiency on maternal diabetes-mediated animal behaviors. The *RORA* wild type (WT) or RORA null (*RORA*^−/−^) background were used to generate either control (CTL) or STZ-induced diabetic (STZ) pregnant dams, and the subsequent 7-8 weeks old male offspring were used for animal behavior analysis. We first measured anxiety-like behaviors, and the results showed that mice from the maternal diabetic (STZ/WT) group buried a significantly reduced number of marbles during the marble-burying test (MBT) (see Fig. 5a) and spent less time in the Open Arm and more time in the Closed Arm in EPM tests compared to the control (CTL/WT) group (see Fig. 5b), while prenatal RORA deficiency (*RORA*^−/−^) had no effect on these behaviors. We then evaluated ALBs, and the results showed that prenatal *RORA* deficiency had no effect on maternal diabetes-mediated decreased ultrasonic vocalization (USV) (see Fig. 5c). Furthermore, mice in the maternal diabetic (STZ/WT) group spent significantly less time in Sniffing, Mounting, and Total Interacting time during the Social Interaction (SI) Tests (see Fig. 5d) compared to the CTL/WT group. Prenatal *RORA* deficiency either mimicked or slightly potentiated this effect, with significantly less time in Sniffing and Total Interacting time during SI test in the STZ/RORA^−/−^ group. In addition, mice from the maternal diabetic (STZ/WT) group spent significantly more sniffing time in the Empty side in the sociability test (see Fig. 5e) and less sniffing time in the Stranger 2 side in the social novelty test compared to the CTL/WT group (see Fig. 5f). Prenatal *RORA* deficiency mimicked this effect, as mice spent significantly less sniffing time on the Stranger 1 side and more time on the Empty side in the sociability test. Additionally, they spent more time on the Stranger 1 side and less time in the Stranger 2 side in the social novelty test in both the CTL/RORA^−/−^ and STZ/RORA^−/−^ groups. In order to eliminate any potential confounding variables, we evaluated the total motor activity of the treated mice and the results showed no significant changes (see Supplementary Fig. 7). Finally, we evaluated the cellular and molecular autism-related phenotypes, including synaptophysin (SYP) expression and spine density. The results showed that maternal diabetes (CTL/WT) significantly decreased SYP expression, including mRNA (see Fig. 5g) and protein (see Fig. 5h, i, Supplementary Fig. 1d) levels, as well as spine density (see Fig. 5j, k), compared to the control (CTL/WT) group, and prenatal *RORA* deficiency (*RORA*^−/−^) treatment mimicked this effect. We conclude that prenatal *RORA* deficiency partly mimics maternal diabetes-mediated ALB, while it has little effect on anxiety-like behavior in offspring.

### In vivo mouse study: postnatal expression of RORA in the amygdala reverses maternal diabetes-induced gene expression and oxidative stress in offspring, while RORA knockdown mimics this effect

**Fig. 6:**
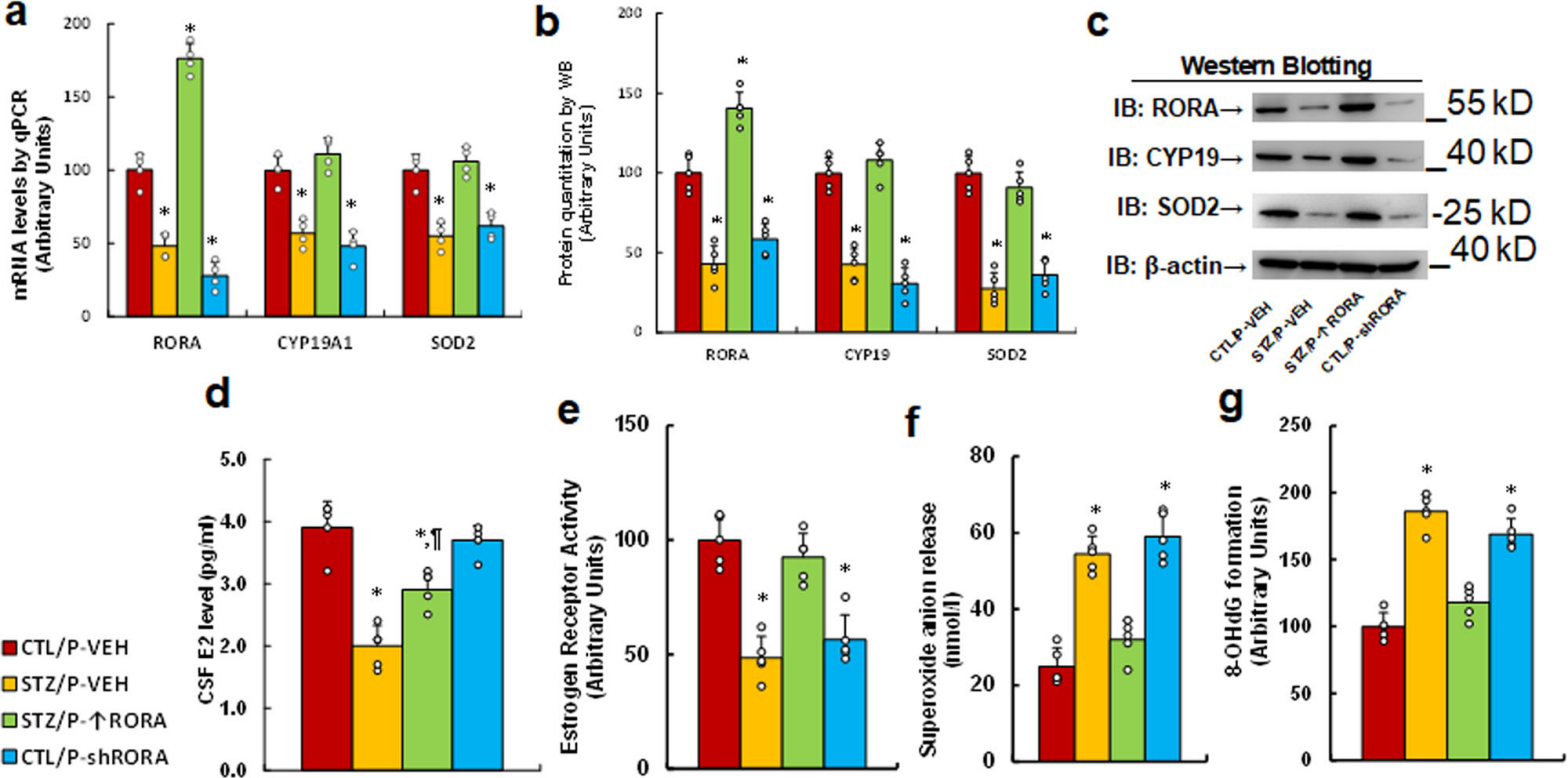
Postnatal expression of RORA in the amygdala reverses maternal diabetes-induced gene expression and oxidative stress in offspring, while RORA knockdown mimics this effect. The male offspring from either the control (CTL) or maternal diabetes (STZ) groups received either vehicle (P-VEH), or lentivirus infusion for either RORA expression (P- ↑ RORA) or RORA knockdown (P-shRORA) at 6 weeks old, and the male offspring were sacrificed for further biomedical analysis at 8 weeks old. **a**–**d** The amygdala tissues were isolated for further analysis as described as follows: **a** mRNA levels by qPCR, *n* = 4. **b** The quantitation of protein levels, *n* = 5. **c** The representative pictures for western blotting. **d** CSF E2 levels, *n* = 5. **e** Estrogen receptor activity, *n* = 5. **f** In vivo superoxide anion release, *n* = 5. **g** The amygdala tissues were isolated for the measurement of 8-OHdG formation, *n* = 5. *, *P* < 0.0001, vs. CTL/P-VEH group; ¶, *P* = 0.002, vs. STZ/P-VEH group. Data were expressed as mean ± SEM.

We evaluated the potential effect of postnatal expression of RORA in the amygdala on maternal diabetes-mediated gene expression and oxidative stress. Male offspring from either the control (CTL) or maternal diabetes (STZ) groups received either vehicle (P-VEH) or lentivirus infusion for either RORA expression (P- ↑ RORA) or RORA knockdown (P-shRORA) at 6 weeks old, and the male offspring were sacrificed for further biomedical analysis at 8 weeks old. We first evaluated gene expression in the amygdala, including mRNA (see Fig. 6a) and protein levels (see Fig. 6b, c, Supplementary Fig 1e), and found that maternal diabetes (STZ/P-VEH) significantly decreased the expression of RORA, CYP19A1, and SOD2 compared to the control (CTL/WT) group. Postnatal manipulation of RORA in the amygdala by lentivirus infusion was successful, with significantly increased *RORA* mRNA resulting from RORA expression lentivirus (STZ/P- ↑ RORA) and decreased *RORA* mRNA as a result of RORA knockdown lentivirus (CTL/P-shRORA). Furthermore, RORA expression (STZ/P- ↑ RORA) reversed, while RORA knockdown (STZ/P-shRORA) mimicked, maternal diabetes (STZ/P-VEH)-mediated suppression of CYP19A1 and SOD2. We then evaluated mRNA expression in the hypothalamus (see Supplementary Fig. 8a) and hippocampus (see Supplementary Fig. 8b). The results showed that the expression of *RORA* and *CYP19A1* was significantly decreased in maternal diabetes (STZ/WT) group compared to the CTL/P-VEH group. Manipulation of RORA expression in the amygdala had no effect, and *SOD2* mRNA levels showed no change in either the hypothalamus or the hippocampus. We also evaluated the potential effect of RORA expression on E2 levels and ER activity. The results showed that maternal diabetes (STZ/P-VEH) significantly decreased CSF estradiol (E2) levels (see Fig. 6d) and ER activity in the amygdala (see Fig. 6e), while RORA expression in the amygdala (STZ/P- ↑ RORA) completely reversed, while RORA knockdown in the amygdala (CTL/P-shRORA) mimicked, maternal diabetes-mediated ER activity, but had little effect on CSF E2 levels. Finally, we evaluated oxidative stress. The results showed that maternal diabetes (STZ/P-VEH) significantly increased superoxide anion release (see Fig. 6f) and 8-oxo-dG formation (see Fig. 6g) to 216% and 186%, respectively, compared to the control (CTL/P-VEH) group; RORA expression (STZ/P- ↑ RORA) reversed, while RORA knockdown (STZ/P- ↑ RORA) mimicked, maternal diabetes (STZ/P-VEH)-mediated oxidative stress. We conclude that postnatal expression of RORA in the amygdala reverses maternal diabetes-induced gene expression and oxidative stress in offspring, while RORA knockdown mimics this effect.

### In vivo mouse study: postnatal manipulation of RORA in the amygdala partly modulates maternal diabetes-induced ALB but has little effect on anxiety-like behavior in offspring

**Fig. 7:**
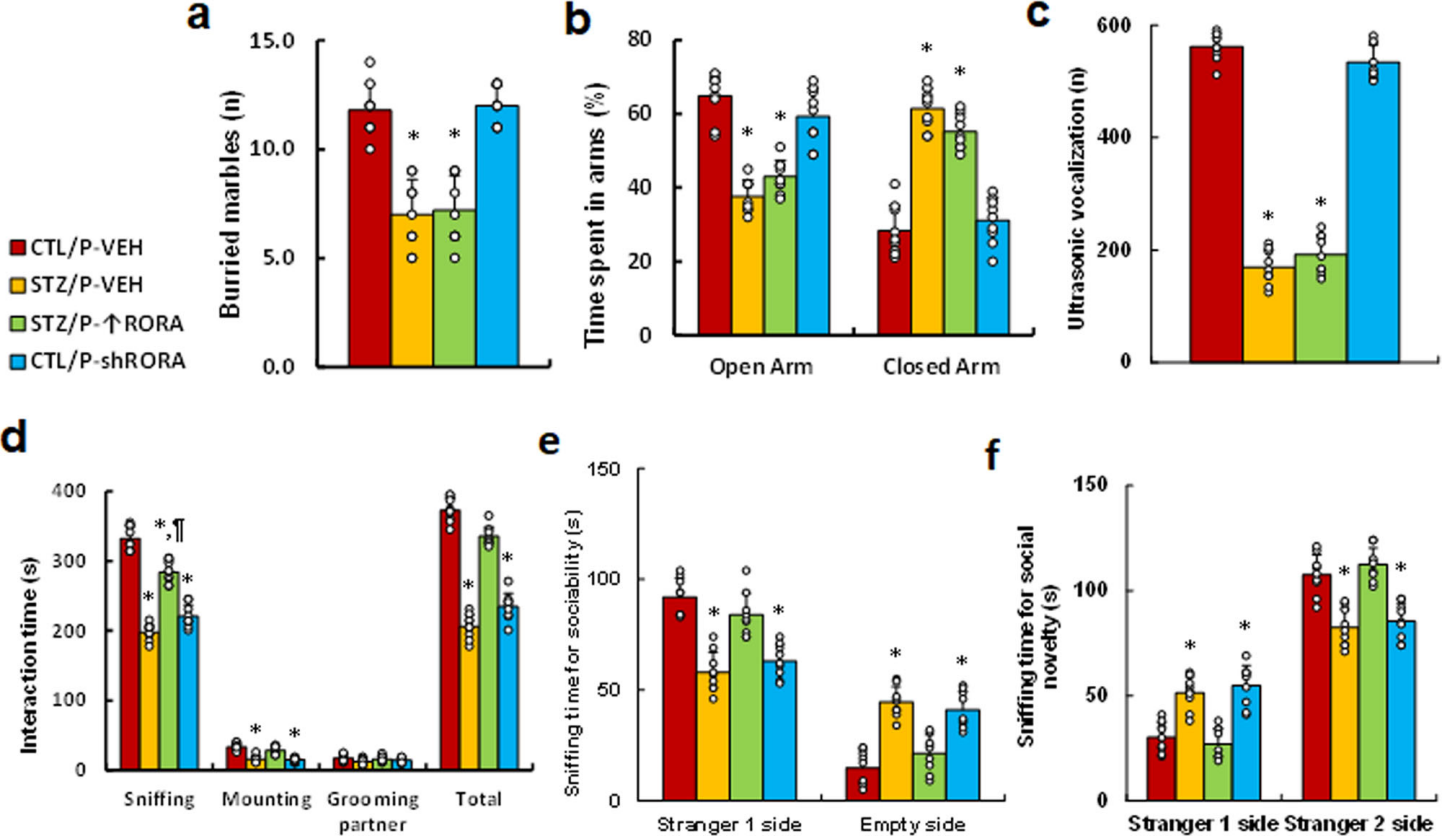
Postnatal manipulation of RORA in the amygdala partly modulates maternal diabetes-induced autism-like behavior but has little effect on maternal diabetes-induced anxiety-like behavior in offspring. Male offspring from either the control (CTL) or maternal diabetes (STZ) groups received either vehicle (P-VEH) or lentivirus infusion for either RORA expression (P- ↑ RORA) or RORA knockdown (P-shRORA) at 6 weeks old, and the male offspring were used for animal behavior analysis. **a** Marble-burying test (MBT), *n* = 9. **b** Time spent in the Open Arm and Closed Arm in the EPM test, *n* = 9. **c** Ultrasonic vocalization, *n* = 9. **d** Social interaction (SI) test, the time spent following, mounting, grooming, and sniffing any body parts of the other mouse was calculated, *n* = 9. **e**, **f** Three-chambered social tests, *n* = 9. **e** Time spent in the chamber for sociability. **f** Time spent in the chamber for social novelty. *, *P* < 0.0001, vs. CTL/P-VEH group; ¶, *P* = 0.0001, vs. STZ/P-VEH group. Data were expressed as mean ± SEM.

We evaluated the potential effect of postnatal manipulation of RORA on maternal diabetes-mediated animal behaviors. Male offspring from either the control (CTL) or maternal diabetes (STZ) groups received either vehicle (P-VEH) or lentivirus infusion for either RORA expression (P- ↑ RORA) or RORA knockdown (P-shRORA) at 6 weeks old, and the male offspring were used for animal behavior analysis. We first measured anxiety-like behaviors, and the results showed that mice from the maternal diabetic (STZ/P-VEH) group buried a significantly reduced number of marbles during the MBT (see Fig. 7a) and spent less time in the Open Arm and more time in the Closed Arm during the EPM tests compared to the control (CTL/P-VEH) group (see Fig. 7b); postnatal RORA manipulation had no effect on these behaviors. We also evaluated ALBs, and the results showed that postnatal RORA manipulation had no effect on maternal diabetes-mediated decreased USV (see Fig. 7c). Furthermore, mice from the maternal diabetic (STZ/P-VEH) group spent significantly less time in Sniffing, Mounting, and Total Interacting time during the SI tests (see Fig. 7d) compared to the CTL/P-VEH group; postnatal RORA expression (STZ/P- ↑ RORA) completely reversed, while RORA knockdown (CTL/P-shRORA) mimicked, maternal diabetes (STZ/P-VEH)-mediated effect. In addition, mice from the maternal diabetic (STZ/P-VEH) group spent significantly more time in the Empty side during the sociability test (see Fig. 7e), and less time in the Stranger 2 side during the social novelty test compared to the CTL/P-VEH group (see Fig. 7f). Again, postnatal RORA expression (STZ/P- ↑ RORA) completely reversed, and *RORA* knockdown (CTL/P-sh*RORA*) mimicked, maternal diabetes (STZ/P-VEH)-mediated effect. In order to eliminate any potential confounding variables, we evaluated the total motor activity of the treated mice and the results showed no significant changes (see Supplementary Fig. 9). We conclude that postnatal manipulation of RORA in the amygdala partly modulates maternal diabetes-induced ALB, but has little effect on anxiety-like behavior in offspring.

### Human study: establishment of ROC curve as an autistic marker based on RORA expression

**Fig. 8:**
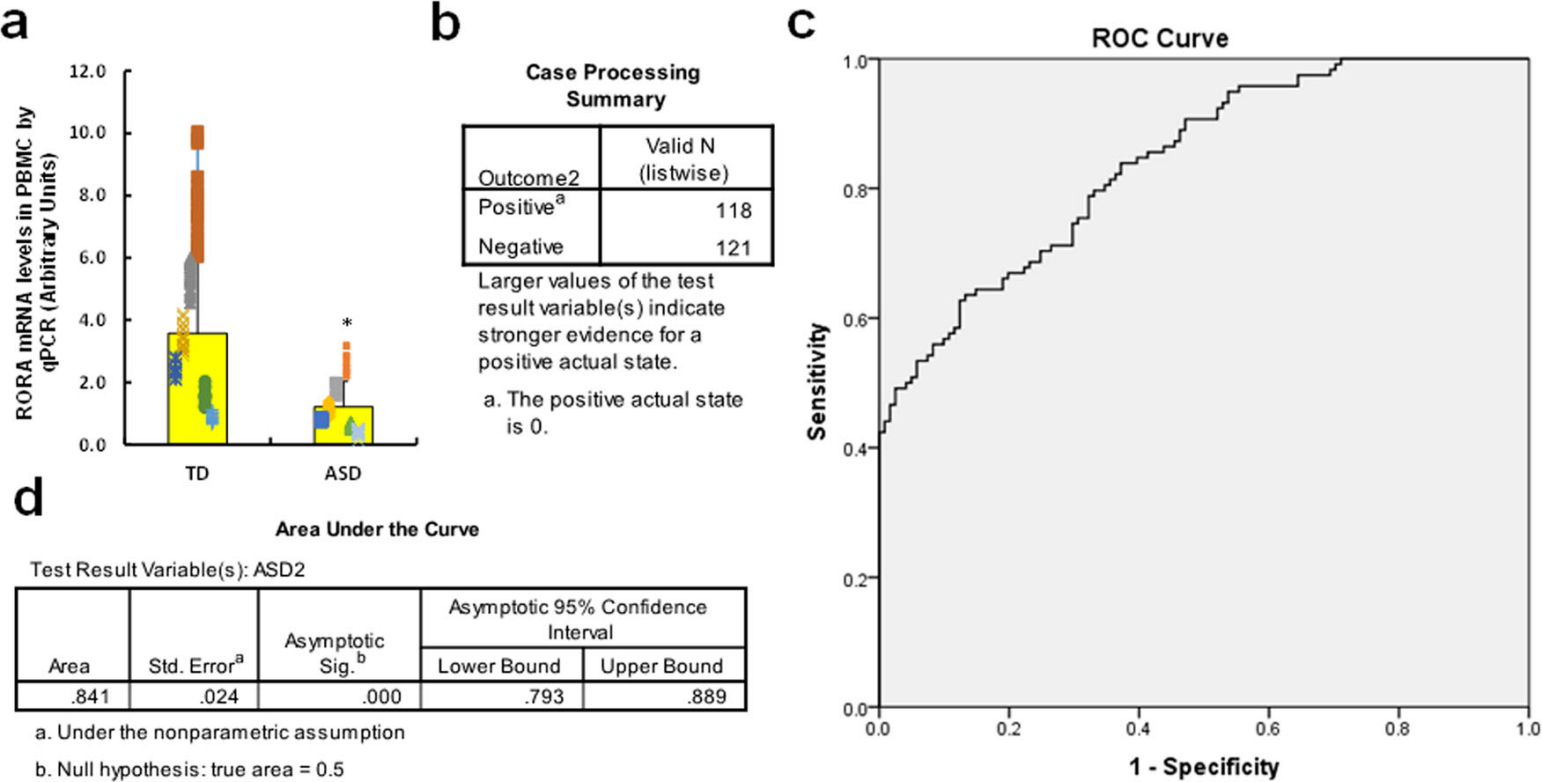
Establishment of ROC curve as an autistic marker based on RORA expression. **a** Three milliliter of peripheral blood was withdrawn from either typical developing (TD, *n* = 118) and ASD (*n* = 121) children (2–6 years old), and the PBMC were isolated for RORA mRNA analysis through real-time quantitative PCR. **b**–**d** The RORA mRNA levels were used to draw the ROC (receiver operating characteristic) curve. **b** Case processing summary, positive represents TD cases, and negative represents ASD cases. **c** ROC curve. **d** Area under the curve. *, *P* < 0.0001, vs. TD group. Data were expressed as mean ± SEM.

We established an autistic marker for screening of ASD patients based on the RORA mRNA levels in PBMC. 3 ml of peripheral blood were withdrawn from either TD (*n* = 118) or ASD (*n* = 121) children (2–6 years old), and the PBMC were isolated for *RORA* mRNA analysis using qPCR. We found that *RORA* mRNA levels in the ASD group decreased to 34.2% compared to the TD group, with a difference of 2.347 and 95% confidence interval of (1.888–2.806), *p* < 0.000 (see Fig. 8a). We also evaluated histone modifications on the *RORA* promoter, and the results showed that there was a significantly increased amount of epigenetic changes on H3K9me3 and H3K27me2 in the ASD group, while there was no significant effect on H3K9me2 and H3K27me3, compared to the TD group (see Supplementary Fig. 10). In order to establish the Pass/Fail Cut/Off value for the screening of ASD patients, the ROC (Receiver Operating Characteristic) curve was established by SPSS 22 software using *RORA* mRNA levels (see Fig. 8b–d). Totally, 118 cases of TD children (considered as positive) and 121 cases of ASD children (considered as negative) were used for calculations (see Fig. 8b), and the ROC curve is shown in Fig. 8c. The area under the curve was calculated to be 0.841 (see Fig. 8d), showing good sensitivity and specificity for ASD screening. We then established the Pass/Fail Cut/Off value for ASD screening as an autistic marker using the coordinates of the curve. As shown in Supplementary Fig. 11, the Pass/Fail Cut/Off value was set as 0.53055 for *RORA* mRNA level with 100% sensitivity and 80% specificity. We concluded that a RORA mRNA level value of <0.53055 could be a potential indicator of ASD in patients.

### Schematic model for maternal diabetes-mediated RORA suppression and contribution to ASD development

**Fig. 9:**
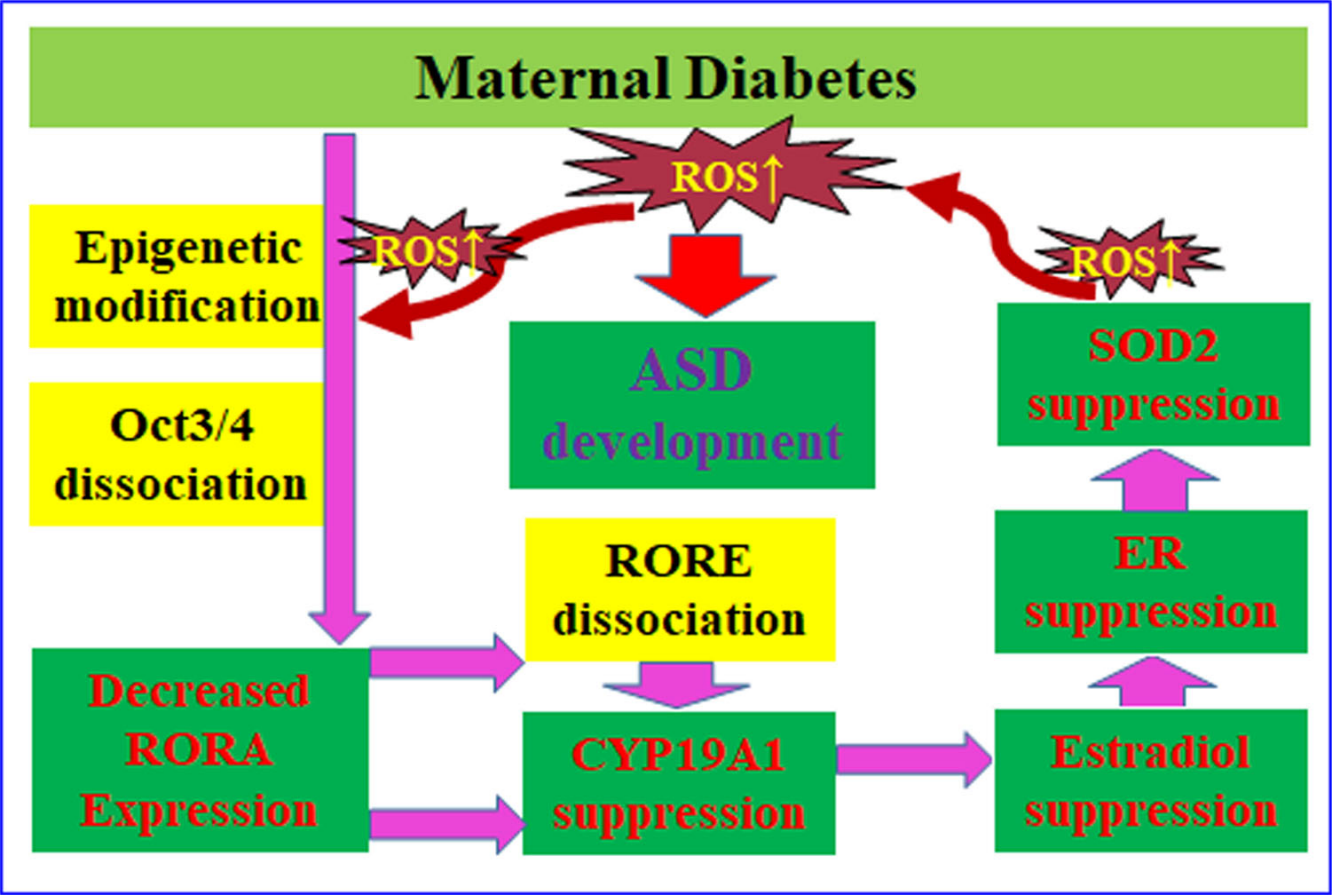
Schematic model for maternal diabetes-mediated RORA suppression and contribution to ASD development. Abbreviations: ASD autism spectrum disorders, CYP19A1 cytochrome P450 family 19 subfamilies A member 1, ER estrogen receptor, Oct3/4 octamer-binding transcription factor-3/4, RORA retinoic acid-related orphan receptor alpha, RORE retinoic acid-related orphan receptor elements, ROS reactive oxygen species, SOD2 superoxide dismutase 2.

We established a schematic model for maternal diabetes-mediated ASD development through RORA suppression (see Fig. 9). Maternal diabetes-mediated ROS over-generation^24^ triggers histone methylation with subsequent dissociation of Oct3/4 from the *RORA* promoter, resulting in decreased RORA expression; RORA suppression induces CYP19A1 suppression through dissociation of RORA with RORE elements on the *CYP19A1* promoter, resulting in decreased estradiol synthesis and estrogen receptor (ER) activity; suppressed E2/ER activity triggers SOD2 suppression through ERE on the *SOD2* promoter with subsequent ROS generation^21^, forming a positive feed-forward loop for ROS generation and oxidative stress and resulting in ASD development in offspring^22^.

## Discussion

In this study, we show that transient hyperglycemia-mediated oxidative stress suppresses the expression of RORA, CYP19A1, and SOD2; prenatal *RORA* deficiency mimics maternal diabetes-mediated ALB, and postnatal RORA manipulation in the amygdala modulates maternal diabetes-mediated ALB in offspring. Thus, *RORA* mRNA level in PBMC could be a potential autistic marker for screening of ASD. Additionally, only male offspring were selected in this study to avoid possible interference from estrogen in female offspring^22,25,26^.

We have previously found that prenatal progestin exposure suppresses ERβ expression in the amygdala instead of other brain areas and subsequently contributes to ALB in offspring, with male offspring being more susceptible than females, and ERβ overexpression in the amygdala significantly restores prenatal progestin exposure-induced ALBs^8,27^. In addition, maternal diabetes-induced autism-like offspring show significant SOD2 suppression in the amygdala^22^, which plays a major role in impacting prenatal dihydrotestosterone exposure-induced autism-like offspring^7^. In this study, we showed that the amygdala plays an important role in the maternal diabetes-mediated neurodevelopmental disorder. To eliminate potential bias, we also evaluated the potential effect of maternal diabetes on other brain regions, such as the hypothalamus and hippocampus (see Supplementary Fig. 5), as well as the cerebral cortex, ventral striatum, and cerebellum (see Supplementary Fig. 6). Most of these regions showed either null results or little effect^28^. Based on the above findings, the amygdala was chosen as a region of interest in this study. It is important to emphasize that the amygdala plays a major role in maternal diabetes-mediated ALBs, but this does not exclude potential minor effects from other regions, which can be reflected in that the overexpression of either SOD2, ERβ, or RORA can only partly, but not completely, reverse maternal diabetes-mediated abnormal behaviors. In this study, we needed to choose a Cre mouse that could be used to specifically target the amygdala for the knockout of *RORA*. An Otp^Cre^ mouse (obtained from Jackson lab) was crossbred with a *RORA*^fl/fl^ mouse, and the RORA expression was measured for almost all the brain regions. The results showed that Otp^Cre^ significantly knocked down RORA expression in the amygdala, but had little effect on other brain regions (see Supplementary Figs. 5 and 6). We also measured RORA expression across all three parts of the amygdala, including the medial, lateral, and basolateral amygdala, and the results showed that the amygdala-specific RORA^−/−^ null mouse (Otp^Cre^-RORA^fl/fl^) had significantly reduced RORA expression not only in the medial amygdala but also in the lateral and basolateral amygdala (see Supplementary Fig. 4). Additionally, RORA expression was not only reduced in the hypothalamus but was also reduced in the hippocampus (see Supplementary Fig. 5). In conclusion, while the amygdala-specific knockdown mice demonstrated some potential leaking effects in the hypothalamus and hippocampus, this model was able to demonstrate knockdown of *RORA* in the entire amygdala, which was sufficient for the purposes of this study.

Our in vitro study in human neural progenitor cells showed that transient HG exposure induces persistent RORA suppression through oxidative stress and subsequent epigenetic modifications on the *RORA* promoter^22^. This was further supported by in vivo mouse study through maternal diabetes-mediated RORA suppression in brain tissues from autism-like offspring, indicating that hyperglycemia memory may play an important role in maternal diabetes-mediated ASD development^24,29^. In addition, we showed that Oct3/4 was dissociated from the *RORA* promoter with subsequent RORA suppression in the presence of transient hyperglycemia, indicating that Oct3/4 may play an important role in neural development by regulation of RORA expression, which is consistent with previous findings^30^. During the in vitro cell culture experiments, we tried to use physiological brain glucose concentrations that have been reported in the range of 1.0–3.0 mM^31^ to establish the hyperglycemia model in human neural progenitor cells, but cells grew very slow and did not look healthy in those situations, and we were not able to achieve enough cells for the experiments. Also, many others have reported that the in vitro neural cell culture requires glucose concentrations >15 mM, which are much higher than normal physiological levels^31,32^. We thus chose 5 mM glucose as the low glucose condition, similar to that used by other researchers^33,34^, and chose 25 mM as the HG treatment^22^. We agree that our in vitro study has potential limitations by using glucose levels that are higher than reported physiological brain levels, but this does not invalidate our findings. In fact, our hyperglycemia-mediated results are indeed meaningful and match our clinical findings. For instance, our in vitro cell study showed that hyperglycemia induces epigenetic changes on the *RORA* promoter with increased H3K9me3 modifications (see Fig. 2e), and our human study showed that PBMC from autistic children has significant epigenetic changes with histone 3 modifications on the *RORA* promoter compared to the TD group (see Supplementary Fig. 7), even though the epigenetic modification spectrum is slightly different between the two. This can be explained by the hypothesis that many other factors, such as prenatal exposure of progestin and androgen as we reported previously^7,8,27^, can also trigger epigenetic changes on the *RORA* promoter. In addition, we also recognize that sometimes, the in vitro cell culture condition must be different with in vivo conditions. For instance, real maternal diabetes exposure usually lasts the entire 10-month pregnancy period, while our cell culture hyperglycemia condition can only achieve a maximum of 5 days.

Our results showed that RORA deficiency had little effect on maternal diabetes-mediated anxiety-like behaviors, as measured in the MBT and elevated plus maze (EPM) tests. Additionally, it had little effect on USV, while it mimicked or slightly potentiated maternal diabetes-mediated SI behaviors and completely mimicked maternal diabetes-mediated social behaviors through three-chambered social tests. More interestingly, RORA deficiency in the amygdala completely mimicked maternal diabetes-mediated reduced synaptophysin expression and spine density in amygdala neurons. In general, our results show that RORA deficiency is the key mediator of maternal diabetes, and it seems indeed to be partly, but not completely, the downstream target, which may be due to the following reasons: a. Our mouse model for neuron-specific RORA deficiency has a potential leaking effect, as RORA was not only knocked down in the amygdala but was also knocked down in the hypothalamus and hippocampus in our *RORA*^−/−^ mice (see Supplementary Fig. 4). b. Our previous findings showed that the amygdala plays a major role in maternal diabetes-mediated ALBs, while this does not exclude other potential minor factors. c. RORA deficiency did not show any effect on maternal diabetes-mediated USVs, which may be because this test was conducted on postnatal day 7, which may have been too early for this kind of mouse model. d. Our results showed that RORA deficiency slightly potentiated maternal diabetes-mediated SI behaviors, which may be because RORA deficiency further worsened oxidative stress in brain tissues as reflected in Fig. 4. In addition, our recent preliminary study showed that RORA may either directly or indirectly regulate the expression of Shank3^35^ and NLGN3^36^. Additionally, maternal diabetes-mediated offspring showed reduced expression of Shank3 and NLGN3 in brain tissues, which further supports our opinion that RORA may play an important role in ASD development, and further investigation is still in process. In addition, we were using 35 mg/kg of STZ for the development of diabetic dams, there is a potential for STZ to exert an effect on fetal development for the subsequent offspring, while during our procedure, a minimum 1 week was arranged for dams to develop into diabetes, and then the blood glucose was measured continuously for 3 days to confirm diabetes before mating with males. In total, there was at least a 10-day gap in time between STZ delivery and mating. In our opinion, by the time 10 days passed after injection, almost all the STZ would have either been degraded or detoxified by the diabetic dams, and the subsequent fetus would have no chance to be directly exposed to any STZ.

It has been reported that CYP19A1 is regulated by RORA either directly or indirectly, contributing to sex bias in ASD development^15,20,23^, while the detailed mechanism remains unclear. Here, we show that RORA directly regulates CYP19A1 expression through a RORE element located at −183 on the *CYP19A1* promoter, which is different from the previous findings^37^; this difference may be due to different cell types. In addition, our results showed that RORA overexpression increased, while *RORA* knockdown decreased, SOD2 expression; while our ChIP analysis data showed that there was no confirmed RORE element with which RORA could interact on the *SOD2* promoter, indicating that SOD2 may be regulated by RORA indirectly. Additionally, it has been reported that SOD2 can be regulated directly by the E2/ER signaling pathway through the ERE element on the *SOD2* promoter^21^. As CYP19A1 controls the conversion of androgen into E2, we suppose that RORA may regulate SOD2 expression indirectly through the CYP19A1/E2/ER signaling pathway. Our study reveals a potential role for RORA-mediated sex bias in ASD development through modulation of CYP19A1/E2-mediated SOD2 expression and oxidative stress in neurons^22,23^. In addition, it has been reported that transient hyperglycemia induces persistent epigenetic changes and altered gene expression during subsequent normoglycemia, which is called hyperglycemia memory^24^. Furthermore, the potential driving force for hyperglycemia memory has been identified to be hyperglycemia-mediated ROS generation and oxidative stress, and overexpression of SOD2 could restore this^24,38^. In this study, SOD2 was overexpressed by SOD2 lentivirus infection to diminish ROS generation and subsequently reverse hyperglycemia/oxidative stress-mediated epigenetic changes on the *RORA* promoter.

Our in vitro study showed that transient hyperglycemia induces persistent RORA suppression in human neural progenitor cells, and the in vivo mouse study showed that maternal diabetes induces RORA suppression in neurons in autism-like offspring. In addition, the human study showed that levels of *RORA* mRNA in PBMC from ASD patients were decreased to 34.2% compared to the TD group. The area under the curve reached 84.1%, showing good sensitivity and specificity for ASD screening and indicating that *RORA* mRNA levels in PBMC may be a potential autistic marker for the diagnosis of ASD. This study has potential limitations for its clinical application due to limited cohort, while a power with *n* = 118 should be qualified to make a conclusion for RORA as an ASD biomarker, another much bigger clinical investigation is currently in our progress to establish a more accurate Pass/Fail Cut/Off value for ASD diagnosis. In addition, our results suggest that in the presence of prenatal exposure of risk factors (e.g., maternal diabetes)^22,25^, RORA expression is not only suppressed in neurons but is also suppressed in PBMC; this can be explained by the hypothesis that maternal diabetes-induced RORA suppression in hematopoietic stem cells during embryonic development can be inherited in PBMC as reported previously^39^. Furthermore, we have previously reported that prenatal hormone exposure, such as progestin and androgen^6–8,27^, is associated with ASD development^40^. We suppose that these factors may also result in RORA suppression in both neurons and PBMC, and a related investigation into this hypothesis is currently in our progress.

## Methods

### Reagents and materials

Neural Progenitor Cells (NPC) ACS-5003 (obtained from ATCC) were cultured in NPC medium with related supplements and antibiotics. All cells were maintained in a humidified incubator with 5% CO_2_ at 37 °C. In some experiments, the ACS-5003 neurons were conditionally immortalized by a hTERT lentivirus vector with an extended life span to achieve higher transfection efficiency and experimental stability^41,42^.

Antibodies for β-actin (sc-47778, 1:2000), C/EBPα (sc-365318, 1:2000), CYP19 (sc-374176, 1:2000), NF1 (sc-74444, 1:2000), Oct-3/4 (sc-5279, 1:2000), Pit1 (sc-393943, 1:2000), PU.1 (sc-390405, 1:2000), RORA (sc-518081, 1:1000), RXRα (sc-515929, 1:2000), SOD2 (sc-137254, 1:2000), Sp1 (sc-17824, 1:2000), SRF (sc-25290, 1:2000) and SYP (sc-17750, 1:1000) were obtained from Santa Cruz Biotechnology. Antibody for 8-oxo-dG (4354-MC-050, 1:2000) was purchased from Novus Biologicals; NeuN (#24307) was purchased from Cell Signaling. Antibodies for acetyl-histone H4 K5, K8, K12, and K16 (H4K5,8,12,16ac, #PA5-40084, 1:2000) were obtained from Invitrogen. Antibodies for histone H3 acetyl K9, K14, K18, K23, K27(H3K9,14,18,23,27ac, ab47915, 1:2000), H4K20me1 (ab9051, 1:2000), H4K20me3 (ab9053, 1:2000), H4R3me1 (ab17339, 1:2000), H3K9me2 (ab1220, 1:2000), H3K9me3 (ab8898, 1:2000), H3K27me2 (ab24684, 1:2000) and H3K27me3 (ab6002, 1:2000) were obtained from Abcam, and estrogen receptor activity was measured using the Estrogen Receptor Transcription Factor Assay Kit (ab207203 from Abcam) per manufacturers’ instructions. Streptozotocin (STZ, #18883-66-4) were obtained from Sigma. Cerebrospinal fluid (CSF) estradiol (E2) levels were measured in mice using the Estradiol (mouse) ELISA Kit (# K3830-100, from BioVision).

### Construction of human reporter plasmids

Human genomic DNA was prepared from NPC cells. In order to construct *RORA/CYP19A1* reporter plasmids, the *RORA/CYP19A1* gene promoters (2 kb upstream of the transcription start site plus first exon) were amplified from Ensembl gene ID: RORA-201 ENST00000261523.9 (for *RORA)* and CYP19A1-201 ENST00000396402.6 (for *CYP19A1*) by PCR and subcloned into the pGL3-basic vector (# E1751, Promega) using the denoted restriction sites with the following primers: *RORA* forward: 5′- gcgc- acgcgt (MluI) - agt act tgc cct caa gga gct -3′ and *RORA* reverse: 5′- gtac- ctcgag (Xho1)- ctt ctg gct cct tca cct gca -3′; *CYP19A1* forward: 5′-gcgc- acgcgt (MluI) - ggacctatgggaaactaacgt -3′ and *CYP19A1* reverse: 5′- gtac- ctcgag (Xho1)- gcg acg tct gga aga tcc cga -3′. All vectors were verified by sequencing, and detailed information on these plasmids is available upon request.

### Preparation of human RORA/Oct3/4 expression lentivirus

The cDNA for human RORA and Oct3/4 (obtained from Open Biosystems) was subcloned into the pLVX-Puro vector (from Clontech) with the restriction sites of EcoR1 and BamH1 using the following primers: *RORA* forward primer: 5′- gtac - gaattc (EcoR1) - atg gag tca gct ccg gca gcc -3′ and *RORA* reverse primer: 5′- gtac - ggatcc (BamH1) - tta ccc atc aat ttg cat tgc -3′; Oct3/4 forward primer: 5′- gtac – gaattc (EcoR1) - atg gcg gga cac ctg gct tcg-3′ and Oct3/4 reverse primer: 5′- gtac - ggatcc (BamH1) - tca gtt tga atg cat ggg aga -3′. The lentivirus for either RORA or empty control (CTL) was expressed through Lenti-X^™^ Lentiviral Expression Systems (from Clontech) per manufacturers’ instructions.

### Preparation of shRORA/shOct3/4 knockdown lentivirus

The shRNA lentivirus plasmid for human RORA (sc-38868-SH), Oct3/4 (sc-36123-SH), or non-target control (sc-108060) were purchased from Santa Cruz Biotechnology. The related lentivirus for sh*RORA*, sh*Oct3/4*, or empty control (CTL) was expressed through Lenti-X^™^ Lentiviral Expression Systems (from Clontech) per manufacturers’ instructions. The purified and condensed lentivirus was used for in vitro gene knockdown, and knockdown efficiency was confirmed by mRNA reduction of more than 65% compared to the control group using real-time PCR (see Supplementary Table 1).

### RT reaction and real-time quantitative PCR

Total RNA from treated cells was extracted using the RNeasy Micro Kit (Qiagen), and the RNA was reverse transcribed using an Omniscript RT kit (Qiagen). All the primers were designed and verified by agarose gel, and the details were provided in Supplementary Table 1. Real-time quantitative PCR was run on iCycler iQ (Bio-Rad) with the Quantitect SYBR green PCR kit (Qiagen). PCR was performed by denaturing at 95 °C for 8 min, followed by 45 cycles of denaturation at 95 °C, annealing at 60 °C, and extension at 72 °C for 10 s, respectively. β-actin was used as the housekeeping gene for transcript normalization, and the mean values were used to calculate relative transcript levels with the ΔΔCT method per instructions from Qiagen^8,43^.

### Western blotting

Treated cells were lysed in an ice-cold lysis buffer with the addition of a protease inhibitor cocktail (Sigma), and the protein concentration was measured using the Coomassie Protein Assay Kit (Pierce Biotechnology). A certain amount of proteins (20–35 g) were loaded and separated in 10% of sodium dodecyl sulfate-polyacrylamide gel electrophoresis gel and then the proteins were transferred to polyvinylidene fluoride membrane. The membrane was then firstly blotted by primary antibodies overnight, and subsequently incubated with differentially labeled species-specific secondary antibodies, either anti-RABBIT IRDye^™^ 800CW (green) or anti-MOUSE (or goat) ALEXA680 (red) for 2 h. After thorough washing, the membranes were scanned and quantitated using the ODYSSEY Infrared Imaging System (LI-COR, NE)^44^.

### Luciferase reporter assay

Cells were seeded in a 6-well plate and cultured in a complete medium until 80% confluent. Cells were then cotransfected by 3 µg of related reporter plasmid as well as 0.2 µg of pRL-CMV-Luc *Renilla* plasmid (from Promega) for internal transfection efficiency control. After 24 hours of transfection, cells were further treated as indicated, then harvested, and the luciferase activity was determined using Dual-Luciferase^TM^ Assay System (Promega) as per manufacturers’ instructions, and the transfection efficiencies were calculated by *Renilla* plasmid accordingly, and the reporter activities were normalized and calculated^43^.

### Chromatin immunoprecipitation (ChIP)

Cells were treated with 1% formaldehyde for 20 min for crosslinking, then terminated by adding 0.1 M of glycine. Cells were then scraped from plates and sonicated following with brief centrifugation, and the subsequent protein supernatant was used for determination of protein concentrations by Coomassie Protein Assay Kit (Pierce Biotechnology). Totally, 500 µg of protein solutions were incubated with BSA/salmon sperm DNA, preimmune IgG, and a slurry of Protein A Agarose beads for pre-clearing, then the immunoprecipitations were performed by adding the indicated antibodies, BSA/salmon sperm DNA and a 50% slurry of Protein A agarose beads for overnight, and the related inputs and immunoprecipitates were thoroughly washed and eluted, then incubated with 0.2 mg/ml of Proteinase K at 42 °C for 2 hours, then switched to 65 °C for another 6 h to reverse crosslinking. DNA fragments were then extracted by phenol/chloroform followed by ethanol precipitation, and the subsequent DNA solution was used for real-time PCR (qPCR), and a ~150 bp fragment on the related promoters was then amplified using the primers provided in Supplementary Table 1^8,43^.

### Measurement of oxidative stress

The intracellular ROS formation was measured by CM-H2DCFDA-based fluorescence emission. Briefly, treated cells were plated in a 24-well plate and cultured until 80% confluent, then 10 μM of CM-H2DCFDA (Invitrogen) was added and incubated for 45 min at 37 °C, and ROS formation was determined at 485/530 nm of excitation/emission wavelength by using FLx800 microplate fluorescence reader (Bio-Tek), and the results were normalized by a series of H2O2 as standard^43,45^. The 8-OHdG formation was measured using an OxiSelect^™^ Oxidative DNA Damage ELISA Kit (Cat No. STA320, from Cell Biolabs Inc.) per manufacturers’ instructions, and the formation of 8-oxo-dG was determined by immunostaining and quantitated by Image J^43^.

### DNA methylation analysis

We developed a real-time PCR-based method for methylation-specific PCR (MSP) analysis on the human *RORA* promoter according to the previously described method with some modifications^46–48^. Genomic DNA from human #ACS-5003 cells was extracted and purified before then being treated by bisulfite modification using the EpiJET Bisulfite Conversion Kit (#K1461, Fisher). The modified DNA was then amplified using methylated and unmethylated primers for MSP that were designed using Methprimer software (http://www.urogene.org/cgi-bin/methprimer/methprimer.cgi) with the following details: Methylated primer: forward 5’- gag ttt agg agt ttg agg tag tag c -3′, reverse 5′- cct cta atc tat aac aat ttc tcg aa -3′; Unmethylated primer: forward 5′- gtt tag gag ttt gag gta gta gtg a -3′; reverse 5′- tta cct cct cta atc tat aac aat ttc tca -3′. Product size: 245 bp (methylated) and 249 bp (unmethylated); CpG island size: 255 bp; Tm: 64.4 °C. PCR was performed by denaturing at 95 °C for 8 min, followed by 45 cycles of denaturation at 95 °C, annealing at 60 °C, and extension at 72 °C for 10 s, respectively. The final methylation readout was normalized by unmethylated input PCR. Additionally, the methylated PCR products were isolated in agarose gel and the relative band densities were quantitated by Image J. using unmethylated PCR products as input control^49^.

### In vivo mouse experiments

The animal protocol conformed to US NIH guidelines (Guide for the Care and Use of Laboratory Animals, No. 85-23, revised 1996) and was reviewed and approved by the Institutional Animal Care and Use Committee from Foshan Maternity and Child Health Care Hospital.

*Generation of neuron-specific RORA knockout mice*. The *RORA*^fl/fl^ mouse, which has loxP flanking sites targeting exon 3 of the RORA gene, was generated by in vitro fertilization and was obtained for the study as a generous gift from Dr. Haimou Zhang from Hubei University. The Otp^Cre^ mouse (#030557), which expresses Cre recombinase in hypothalamic and medial amygdala neurons, was obtained from Jackson Laboratories. To generate neuron-specific RORA^−/−^ null mice (Otp^Cre^-*RORA*^fl/fl^), *RORA*^fl/fl^ mice were cross-bred with Otp^Cre^ mice for over 4 generations on the C57BL/6J background. Positive offspring were confirmed by genotyping through PCR using specific primers (see Table S1) for the presence of both loxP sites within RORA alleles and Cre recombinase^13,50^.

*Generation of diabetic mice*. All the experimental mice were either *RORA* wild type (WT) or *RORA* null (*RORA*^−/−^) mice with a C57BL/6 J mixed genetic background. In the generation of diabetic mice, adult (3-month-old) female mice with either WT or *RORA*^−/−^ backgrounds were monitored for estrous cycles with daily vaginal smears. Only mice with at least two regular 4 to 5-day estrous cycles were included in the studies. Chronically diabetic female mice were induced by injection of 35 mg/kg streptozotocin (STZ, 0.05 M sodium citrate, pH 5.5) after an 8-h fasting period. Animals were then monitored through measurement of blood glucose for one week after STZ injection for the development of diabetes, and mice with blood glucose >250 mg/dl for 3 days continuously were considered diabetes positive, while control (CTL) mice received only vehicle injection.

*Mouse Protocol 1 for prenatal treatment of diabetes or RORA deficiency*. Verified pregnant dams (*n* = 20 in total) were randomly assigned to the following 4 groups: Group 1: CTL group mice with RORA WT background (CTL/WT); Group 2: STZ mice with *RORA* WT background (STZ/WT); Group 3: CTL group mice with RORA knockdown background (CTL/*RORA*^−/−^); Group 4: STZ mice with *RORA* null background (STZ/*RORA*^−/−^). Neurons from the amygdala were isolated on an embryonic day 18 (E18) as described below. The male offspring were separated from the dams on day 21, fed with normal chow until 7–8 weeks of age, then the mice underwent behavior tests including anxiety-like behaviors and ALBs (see details in Supplemental file Data S1). The offspring were then sacrificed and various brain tissues, including the amygdala, hypothalamus, and hippocampus, were isolated, flash-frozen in dry ice, and then stored in a −80 °C freezer for analysis of gene expression and oxidative stress. CSF was collected as described below^51^ for the measurement of estradiol (E2) levels.

*Mouse Protocol 2 for postnatal manipulation of RORA expression in the amygdala*. Male offspring (6 weeks old, 36 mice in total) from either the CTL or STZ group in Mouse Protocol 1 were anesthetized with a mixture of ketamine (90 mg/kg) and xylazine (2.7 mg/kg) and implanted with a guide cannula targeting the amygdala (26 gauge; Plastics One)^52^. The following stereotaxic coordinates from the bregma were used for the amygdala: anteroposterior (AP) = −1.4, mediolateral (ML) = ±3.5, dorsoventral (DV) = −5.1. Dorsoventral coordinates, which were based on the mouse brain atlas^53^, were measured from the skull surface with the internal cannula extending 2 mm beyond the end of the guide cannula. The cannula was attached to the skull with dental acrylic and jeweler’s screws and closed with an obturator^54^. An osmotic minipump (Alzet model 2002; flow rate 0.5 μl/h; Cupertino, CA) connected to a 26-gauge internal cannula that extended 1 mm below the guide was implanted and used to deliver RORA overexpression (↑RORA), RORA knockdown (shRORA), or vehicle (VEH) lentivirus. Vehicle consisting of artificial CSF (aCSF; 140 mM NaCl, 3 mM KCl, 1.2 mM Na_2_HPO_4_, 1 mM MgCl_2_, 0.27 mM NaH_2_PO_4_, 1.2 mMCaCl_2_, and 7.2 mM dextrose, pH 7.4) was used for the infusion of the lentivirus. Infusion (flow rate 0.5 µl/h) began immediately after placement of the minipump, and 0.5 μl of 2 × 10^3^ cfu total lentiviruses was infused for 1 h. The experimental mice were separated into 4 groups, with *n* = 9 in each group. Group 1: CTL offspring with vehicle control lentivirus infusion (CTL/P-VEH); Group 2: STZ offspring with vehicle control lentivirus infusion (STZ/P-VEH); Group 3: STZ offspring with RORA expression lentivirus infusion (STZ/P- ↑ RORA); Group 4: CTL offspring with shRORA lentivirus infusion (CTL/P-shRORA). Cannula placement was verified histologically postmortem by the injection of 0.5 μl of India ink (volume matching that of drug delivery in the experiments). Six mice whose dye injections were not located in the amygdala were excluded from data analysis (see Supplementary Table 2). Two weeks after lentivirus infusion, the offspring were used for behavior tests followed by biomedical analysis, as indicated in Mouse Protocol 1^8^.

### Animal behavior test

The animal behavior test of offspring was usually carried out at 7–8 weeks of age unless otherwise indicated. Anxiety-like behavior was evaluated using the MBT and the EPM tests^27^. Autism-like behavior was evaluated using USV, SI tests, and a three-chambered social test^55–57^.

*MBT*. The experimental mouse was placed in a clean cage (35 × 23 × 19 cm^3^) that was filled with wood chip bedding (5 cm depth) containing 20 of colored 1 cm-diameter glass marbles with a 5 × 4 arrangement. The number of buried marbles (>50% covered by bedding material) was counted in 30 min by the double-blinded qualified technician^8,27,58,59^.

*EPM*. The EPM test was performed to characterize anxiety-like behavior in mouse offspring. The EPM Package together with IR Beam Detection (Cat #: MED-ELVM-1R) was purchased from Med Associates Inc. The maze has two of open and closed arms, and dual sensors located at the entrance to each goal runway can differentiate between runway exploration and entrance for more accurate position detection. The experimental mouse was placed in the junction area and the movements were quantitated by infrared beams installed on each arm for 5 min, and then automatically recorded by MED-PC software (Cat #: SOF-735, Med Associates), and the time spent in both the open and closed arms was quantitated by a double-blinded technician. In addition, total motor activity was counted in an open-field test during a 30 min assessment^8,54^.

*USV*. The USV test was conducted on postnatal day 7 during a maternal-separation paradigm. The individual pup was isolated and placed in the sound-proof chambers, and the USV was recorded by an externally polarized condenser microphone (30–300 kHz) that was attached 15–20 cm above the floor of the isolation chamber, and the microphone was connected to Avisoft-UltrasoundGate recording software (from Avisoft Bioacoustics) and the pup-emitted calls were transferred to WAV sound files. The recorded number of USV was analyzed by a generalized linear model through a negative binomial distribution and a log-link function^55,56^.

*SI test*. The subjects, including Test and Stranger mice, were habituated in the arena separately for 5 min before the test. During the test, the mice were placed into the apparatus for 20 min and the time spent following, mounting, grooming, and sniffing any body parts of the other mouse was counted as the indicator of social engagement, and the SI time was quantitated and analyzed by EthoVision XT animal tracking software^60^. Each “Stranger” mouse during the test was used only once, and matched with the same gender, weight, and age, and had no previous contact with the test mouse^8,27,58,59^.

*Three-chambered social test*. Totally, 7- to 8-week-old mice were employed to determine the sociability and the preference for social novelty. Target subjects, including Stranger 1 and Stranger 2, were placed inside the wire cages for 3 days before the beginning of testing, and the test mice were placed in the testing room for at least 45 min before the start of behavioral tests. For the sociability test, the test mouse was placed to the middle chamber and left to habituate for 5 min, after which an unfamiliar Stranger 1 mouse was introduced into a wire cage in one of the side-chambers and an empty wire cage on the other side-chamber. The test mouse was allowed to explore freely all 3 chambers for 10 min. Following this, a novel Stranger 2 mouse was introduced into the previously empty wire cage and the test mouse was again left to explore freely for 10 min. All the parameters, including time spent in each chamber, number of entries into the chambers, and track maps, were recorded and calculated by automated SMART software^22,57^.

### Collection of cerebrospinal fluid (CSF)

Procedure for CSF collection is based on a previously established protocol with minor modifications. Briefly, the mouse was anesthetized, under the dissecting microscope, the shaved head was clamped in place for dissection, and the layers of muscles were carefully dissected away using forceps and the dura over the cisterna magna was exposed, this area has large blood vessels running through, which is optimal for capillary insertion and CSF collection. The angle of the glass capillary was carefully adjusted and the sharpened tip of the glass capillary was aligned and eventually tapped through the dura to collect CSF using a micromanipulator control. CSF should automatically be drawn into the capillary tube once the opening has been punctured, and around 20 µl of CSF was collected. The glass capillary was gently removed from the mouse by micromanipulator control, and the CSF was then mixed with 1 µl of 20× protease inhibitor in a 1.5 ml centrifuge tube for quick centrifugation (pulse spin for 5 s at maximal speed), and the CSF samples can be aliquoted for either immediate analysis or stored at −80 °C^51^.

### In vitro primary culture of amygdala neurons

The amygdala was dissected from an embryo in dams on an embryonic day 18 (E18), then the tissues were treated for 15 min at 37 °C by 0.05% of trypsin EDTA, then replaced by soybean trypsin inhibitor (Sigma) to stop the reaction for 5 min at 37 °C. The tissues were then dissociated in supplemented Neurobasal A (Invitrogen) solution and then resuspended in culture media, including Neurobasal A, B27, 1× GlutaMAX, and 100 U/ml Pen/Strep (from Invitrogen), the isolated amygdala neurons were maintained at 37 °C and 5% CO_2_ for further biomedical analysis^61^.

### Dendritic spine density analysis

Dendritic spine density was performed in isolated mature amygdala neurons. Cells were infected by pLenti-GFP lentivirus (#LTV-400 from Cell Biolabs Inc.) for visualization^62^. Different dendritic sections were selected by random and the fluorescent images were taken through a 63× oil immersion objective and 3.5× digital zoom using a confocal microscope (Carl Zeiss, Germany) with the same settings for all samples. The dendritic spine was counted on segments that were 50–100 μm apart from the soma with no other dendrites crossing their trajectory, and all the operations were conducted by double-blinded investigators^63,64^.

### Immunostaining

Treated cells were transferred and incubated on coverslips, then cells were washed by PBS and fixed by 4% of paraformaldehyde for 20 min followed by incubation with 0.3% Triton X-100 in PBS for 15 min. The cells were blocked by normal goat serum, then incubated with 8-oxo-dG anti-mouse antibody (#4354-MC-050, from Novus Biologicals) at 4 °C for overnight, and subsequently incubated with secondary antibody Alexa Fluor 488 for 2 h. The coverslips were then mounted by antifade Mountant with DAPI (for nuclei staining, in blue). The representative pictures were taken using a confocal laser microscope, and the staining was further quantitated by Image J. software.

### In vivo superoxide anion (O_2_^.^^−^) release

Superoxide anion release in amygdala tissues was extracted by dimethyl sulfoxide-tetrabutylammonium chloride (DMSO-TBAC) solution and the TBAC-O_2_^.^^−^ complex was then further detected by use of the luminol-EDTA-Fe enhanced chemiluminescence system^65^. Briefly, the biological tissues were isolated and purged continuously by N2 gas to remove traces of oxygen, and O_2_^.−^ from tissues was extracted by DMSO-TBAC, then the chemiluminescent reagents and O_2_^.−^ extract solutions were pumped into a glass scintillation vial which was placed in the luminometer, and the chemiluminescence intensity was continuously monitored for 2 min and calculated. A series of fresh xanthine solutions (dissolved in 1 mM PBS, pH 7.5) was injected into the apparatus and then merged with xanthine oxidase solution to produce different quantities of superoxide anions and the subsequent chemiluminescent emission showed a linear response to the concentrations of xanthine in a logarithmic graph, and the standard curve was calibrated and established. The superoxide anion release from the sample was calculated from the xanthine/xanthine oxidase reaction standard curve, and the result was expressed as nmol/l^45^.

### Human study protocol

The human study was carried out in accordance with the recommendations of the Human Subject Guidelines of the Human Subjects Institutional Review Board. The protocol was approved by Hainan Women and Children’s Medical Center, and all subjects gave written informed consent in accordance with the Declaration of Helsinki. Each autistic child was rigorously matched with one control subject of the TD group. 121 cases of ASD children and 118 cases of matched TD children (2–6 years old) were ultimately identified after the exclusion of outliers, and participants were included in this study with informed written consent from their parents. Both autistic and TD children were assessed for verbal IQ (intelligence quotient), performance IQ, and full-scale IQ using the Wechsler Intelligence Scale for Children IV (WISC-IV)^66,67^. As a result, children from the autistic and TD groups did not differ significantly in age, sex, total IQ, performance IQ, and verbal IQ (see Supplementary Table 3). ASD diagnosis was based on several clinical assessments by a multidisciplinary team and was further confirmed by licensed clinical psychologists and psychiatrists at Hainan Women and Children’s Medical Center using the DSM-5 (Diagnostic and Statistical Manual of Mental Disorders, Fifth Edition) as the diagnostic criteria^6,66,68^. Totally, 3–5 ml of peripheral blood were withdrawn from the selected children and the PBMCs were isolated from fresh blood using Lymphoprep^TM^ reagents (#07861, from STEMCELL Technologies) for analysis of RORA mRNA expression. The ROC (Receiver Operating Characteristic) curve was established and the Pass/Fail CutOff Value was defined based on RORA mRNA levels using SPSS 22 software as the autistic marker for screening of ASD children^6^.

### Statistics and reproducibility

The data were given as mean ± SEM, and all the experiments were performed at least in quadruplicate unless indicated otherwise. One-way analysis of variance (ANOVA) followed by the Turkey–Kramer test was used to determine the statistical significance of different groups for in vitro cell study. For in vivo mouse study, two-way ANOVA followed by the Bonferroni post hoc test was used to determine the differences of two factors (i.e., RORA deficiency and maternal diabetes) in Mouse Protocol 1, and one-way ANOVA was used for analysis in Mouse Protocol 2. For the human study, the unpaired Student’s t-tests and one-way ANOVA analysis were used to determine the group differences in the analysis of mRNA, age, and ChIP. The gender ratio was examined using the *χ*^2^ test; and ROC (Receiver Operating Characteristic) curve analysis was used to establish the ROC curve and Pass/Fail Cutoff Value using SPSS 22 software, with a *P* value of <0.05 being considered significant^8,69^.

### Reporting summary

Further information on research design is available in the Nature Research Reporting Summary linked to this article.

## Supplementary information

Supplementary Information

Description of Additional Supplementary Files

Supplementary Data 1

Supplementary Data 5

Supplementary Data 4

Supplementary Data 2

Supplementary Data 3

Reporting Summary

## Data Availability

All the data that support this study are available from either the paper or supplementary tables and figures, and the source data for all the figures are provided in Supplementary Data. Supplementary Data 1 contains data for the in vitro cell culture study except for Fig. 1a, b; Supplementary Data 2 contains data for the in vitro cell culture study represented in Fig. 1a, b; Supplementary Data 3 contains data for the in vitro cell culture study for mapping of *RORA*/*CYP19A11* regulatory elements; Supplementary Data 4 contains data for the in vivo mouse study; Supplementary Data 5 contains data for the in vivo human study. All the other information for this study is available from the corresponding author upon reasonable request.
