# Supplementary material for: Maternal diabetes-mediated RORA suppression in mice contributes to autism-like offspring through inhibition of aromatase
Source: Commun Biol. 2022 Jan 13;5:51. doi: 10.1038/s42003-022-03005-8 (PMC8758718; doi:10.1038/s42003-022-03005-8)
Supplement: Supplementary file 1 — Supplementary Information [file 42003_2022_3005_MOESM1_ESM.pdf]

Maternal Diabetes-Mediated RORA Suppression in Mice Contributes to Autism-Like Offspring through Inhibition of Aromatase

Hong Yu[1,*], Yanbin Niu[2,*], Guohua Jia[3,*], Yujie Liang[4], Baolin Chen[1], Ruoyu Sun[1], Min Wang[3], Saijun Huang[1], Jiaying Zeng[1], Jianpin Lu[4], Ling Li[3,#], Xiaoling Guo[1,#], Paul Yao[1,3,#]

Supplemental Information

Supplementary Table 1. Sequences of primers for the real time quantitative PCR (qPCR)

Gene	Species	Analysis	Forward primer (5'→3')	Reverse primer (5'→3')
β-actin	Human	mRNA	gatgcagaaggagatcactgc	atactcctgcttgctgatcca
RORA	Human	mRNA	ggagaagtcagcaaagcaatg	gacattcggccaaattttaca
CYP19A1	Human	mRNA	gactcgagtttttccccaaac	gtgtaacgaggatggctttca
SOD2	Human	mRNA	gcctacgtgaacaacctgaac	tgaggtttgtccagaaaatgc
OCT3/4	Human	ChIP	tcctgaagcagaagaggatca	ccgcagcttacacatgttctt
CYP19A1	Human	ChIP	cacttgctgaggtcttttgct	catgacctcctctggaatgag
RORA	Human	ChIP	cctaggaaagggcatttgaca	ccagccacaaagaatgaggta
β-actin	Mouse	mRNA	tcttgggtatggaatcctgtg	atctccttctgcatcctgtca
RORA	Mouse	mRNA	attggacatcaatgggatcaa	tttggatatgttctgggcaag
CYP19A1	Mouse	mRNA	tatgaacgatccgtcaaggac	ttctctttcgtcaggtctcca
SOD2	Mouse	mRNA	ggcctacgtgaacaatctcaa	tcaggtttgtccagaaaatgg
SYP	Mouse	mRNA	ttcgctttcatgtggctagtt	aagtcacagggtccctcagtt
loxP	Mouse	Genotype	ttgtgtataccaccacaagtgcacc	agtacaggacacttcggtgtctacc
Cre	Mouse	Genotype	cttgggctgccagaatttctc	cccagaaatgccagattacg

Supplementary Table 2. Histological verification record of cannula placement for amygdala injection by postmortem injection of 0.5 μl of India ink

Mouse #	CTL/P-VEH	STZ/P-VEH	STZ/P-↑RORA	CTL/P-shRORA
1	Pass	Pass	Pass	Pass
2	Pass	Pass	Pass	Fail
3	Pass	Pass	Fail	Pass
4	Pass	Pass	Pass	Pass
5	Fail	Pass	Pass	Pass
6	Pass	Pass	Pass	Pass
7	Pass	Pass	Fail	Pass
8	Pass	Fail	Pass	Pass
9	Pass	Pass	Pass	Fail
10	Pass	Pass	Pass	Pass

Supplementary Table 3. Participant characteristics in the ASD case-control study (Mean ± SD)

	Autistic Case group (n=121)	TD Control group (n=118)	Statistic (t or χ^2 value)	*P* value
Age (years)	4.67 ± 1.08	4.84 ± 1.16	0.911 (t-Test)	0.381
Sex (m/f)	102/19	99/19	0.000 (χ^2 test, df=1)	0.876
Full scale IQ	103.14 ± 11.41	104.21 ± 11.62	1.398 (t-Test)	0.154
Performance IQ	102.87 ± 13.57	103.66 ± 9.57	1.047 (t-Test)	0.281
Verbal IQ	102.91 ± 12.49	103.18 ± 11.96	1.149 (t-Test)	0.247

Note: m/f= male/female ratio; df: degrees of freedom; TD= typically developing; IQ= intelligence quotient

Supplementary Figure 1

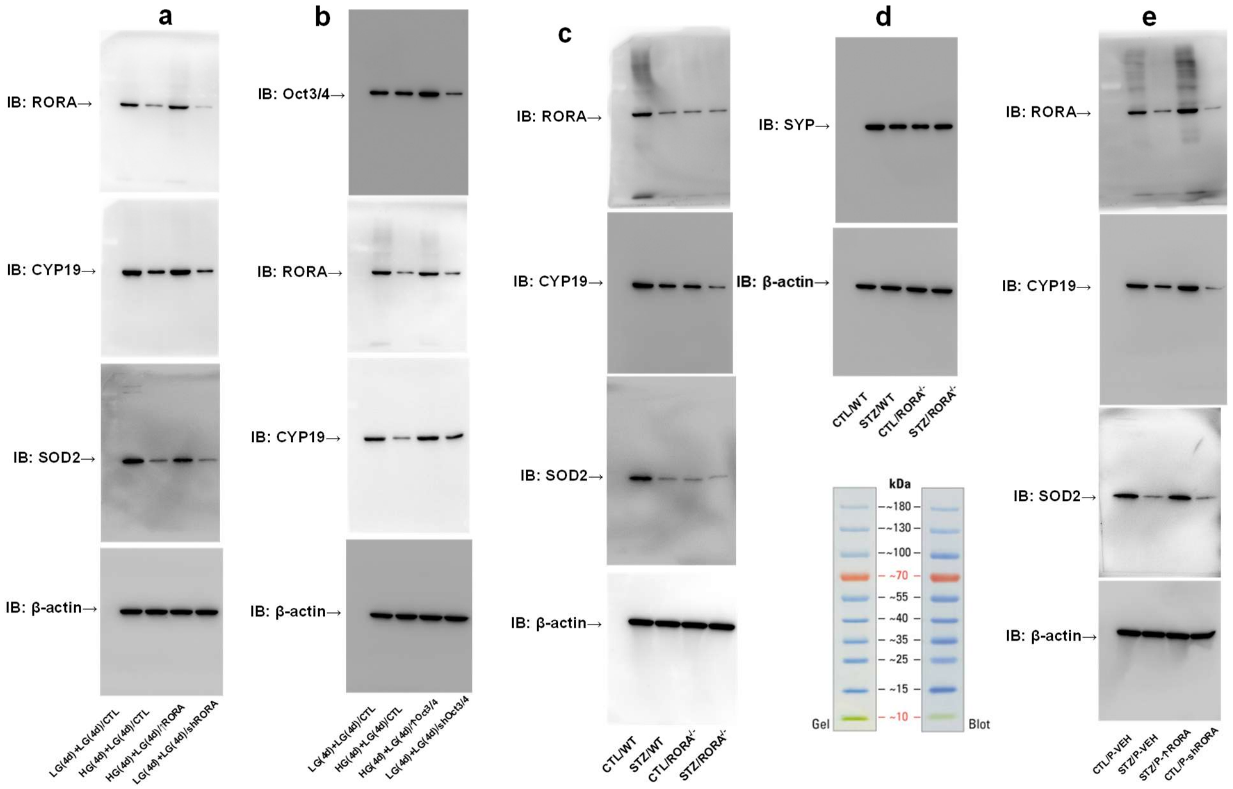

Supplementary Figure 1. Representative pictures of full blots for Western Blotting. (a) Full blots for Figure 1e. (b) Full blots for Figure 2i. (c). Full blots for Figure 4c. (d) Full blots for Figure 5i. (e) Full blots for Figure 6c.

Supplementary Figure 2

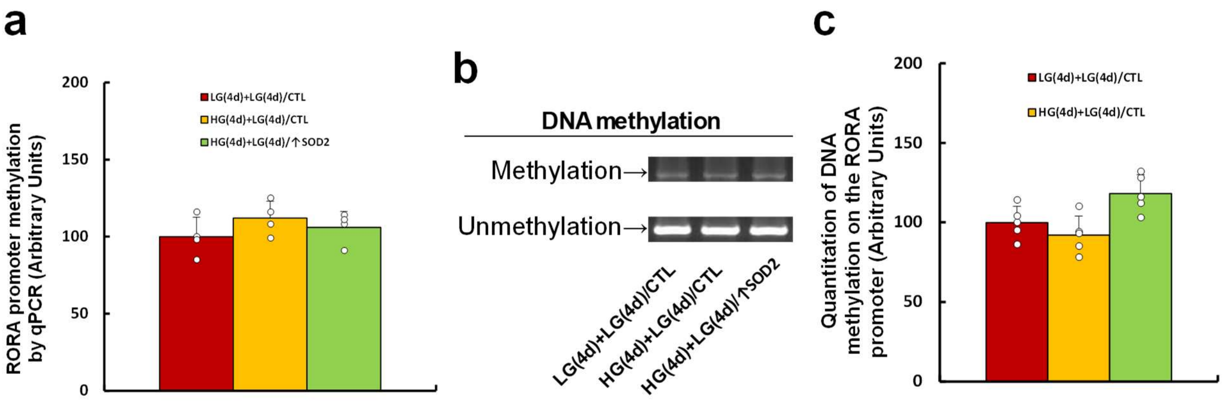

Supplementary Figure 2. Potential effect of hyperglycemia and SOD2 expression on DNA methylation on the RORA promoter. The ACS-5003 neurons were treated by either 4-day LG plus 4-day LG (LG(4d)+LG(4d)), 4-day HG plus 4-day LG (HG(4d)+LG(4d)). The cells were infected with SOD2 lentivirus (HG(4d)+LG(4d)/SOD2↑) on day 4, and the cells were then used for analysis of DNA methylation on the RORA promoter. (a) Quantitation of DNA methylation by qPCR, n=7. (b) Representative pictures for methylation and unmethylation in PCR products. (c) Quantitation of methylation products for (b), n=7. Data were expressed as mean ± SEM.

Supplementary Figure 3

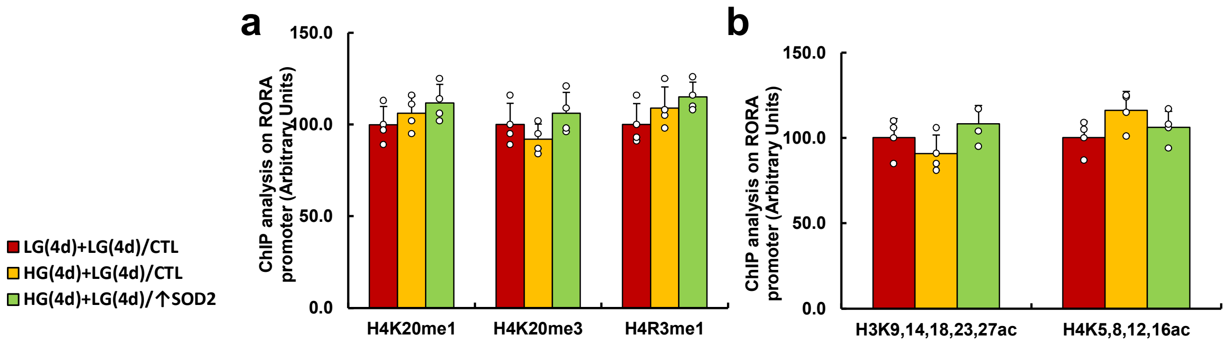

Supplementary Figure 3. Potential effect of hyperglycemia and SOD2 expression on histone modifications on the RORA promoter. The ACS-5003 neurons were treated with either 4-day LG plus 4-day LG (LG(4d)+LG(4d)) or 4-day HG plus 4-day LG (HG(4d)+LG(4d)), or the cells were infected on with SOD2 lentivirus (HG(4d)+LG(4d)/↑SOD2) on day 4. The cells were then used for ChIP analysis. (a) Histone H4 methylation on the RORA promoter, n=4. (b) Histone acetylation on the RORA promoter using H3K9,14,18,23,27ac and H4K5,8,12,16ac antibodies, n=4. Data were expressed as mean ± SEM.

Supplementary Figure 4

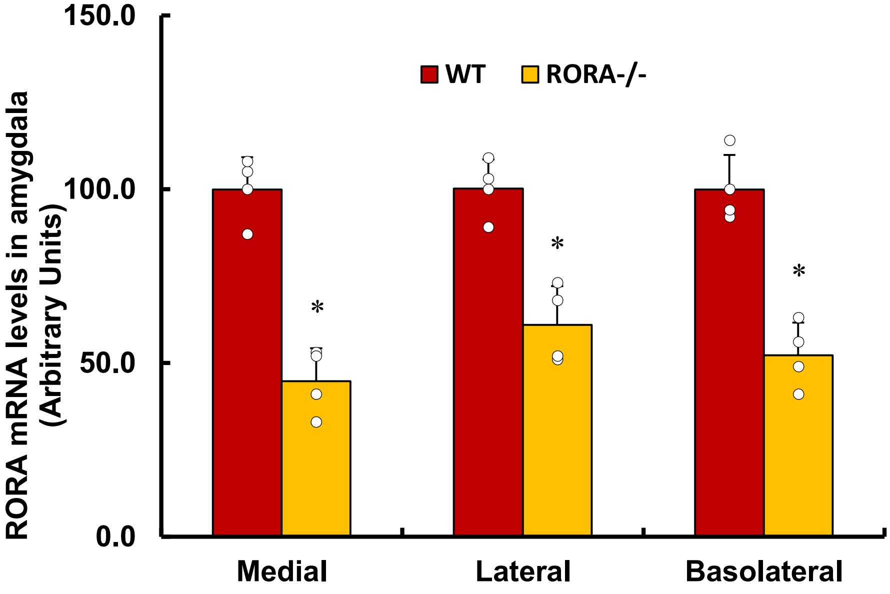

Supplementary Figure 4. Amygdala-specific *RORA*$^{-/-}$ null mice (Otp^{Cre}-*RORA*$^{fl/fl}$) showed decreased RORA expression not only in the medial amygdala, but also in the lateral and basolateral amygdala. *RORA* wild type (WT) or *RORA* null (*RORA*$^{-/-}$) mice were crossbred with Otp^{Cre} mice to generate amygdala-specific *RORA*$^{-/-}$ null mouse (Otp^{Cre}-*RORA*$^{fl/fl}$), and different parts of amygdala, including the medial, lateral and basolateral sections, were isolated for *RORA* mRNA analysis by qPCR, n=4. *, P<0.0001, vs WT group. Data were expressed as mean ± SEM.

Supplementary Figure 5

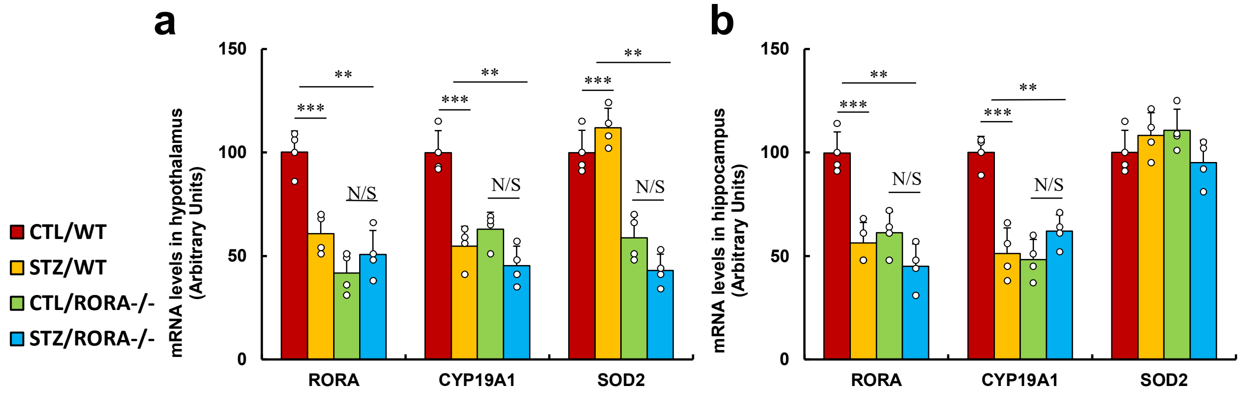

Supplementary Figure 5. Potential effect of prenatal RORA deficiency on maternal diabetes-induced gene expression in offspring. The *RORA* wild type (WT) or *RORA* null ($RORA^{-/-}$) background were used to generate either control (CTL) or STZ-induced diabetic (STZ) pregnant dams. The subsequent 7-8 week-old male offspring were sacrificed and tissues for the hypothalamus and hippocampus were isolated for mRNA analysis. (a) mRNA levels in the hypothalamus, n=4. (b) mRNA levels in the hippocampus, n=4. ***, *P*<0.0001; **, *P*<0.001; N/S, no significance. Data were expressed as mean ± SEM.

Supplementary Figure 6

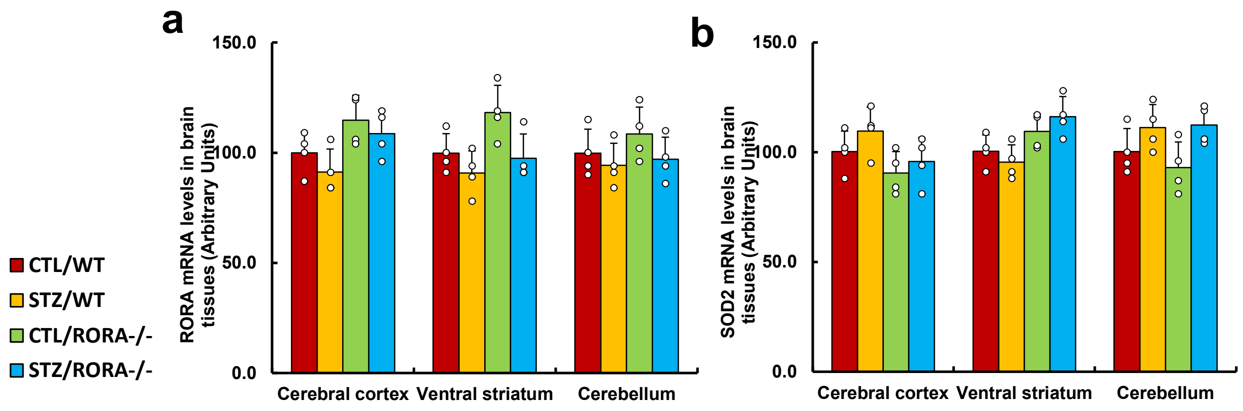

Supplementary Figure 6. Potential effect of prenatal RORA deficiency on maternal diabetes-induced gene expression in offspring. The *RORA* wild type (WT) or *RORA* null (*RORA*$^{-/-}$) background were used to generate either control (CTL) or STZ-induced diabetic (STZ) pregnant dams. The subsequent 7-8 week-old male offspring were sacrificed and the tissues for the cerebral cortex, ventral striatum and cerebellum were isolated for mRNA analysis. (a) mRNA levels for *RORA*, n=4. (b) mRNA levels for *SOD2*, n=4. Data were expressed as mean ± SEM.

Supplementary Figure 7

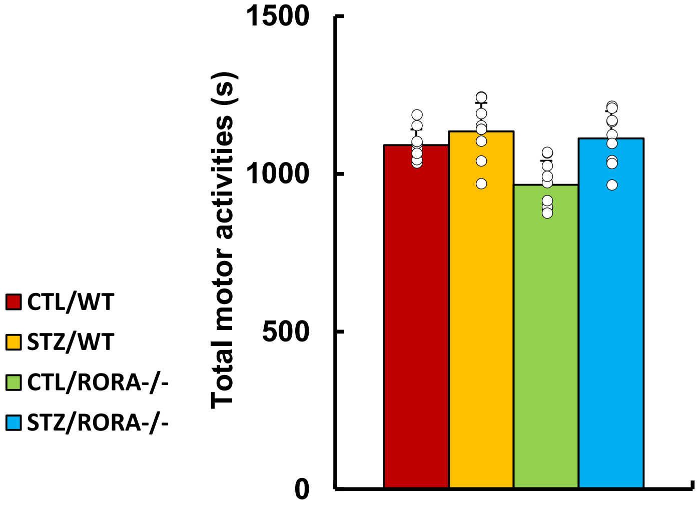

Supplementary Figure 7. Prenatal RORA deficiency and maternal diabetes have no significant effect on total motor activity in offspring. The *RORA* wild type (WT) or *RORA* null ($RORA^{-/-}$) background mice were used to generate either control (CTL) or STZ-induced diabetic (STZ) pregnant dams, and the subsequent 7-8 week-old male offspring were used for analysis of total motor activity, n=9. Data were expressed as mean ± SEM.

Supplementary Figure 8

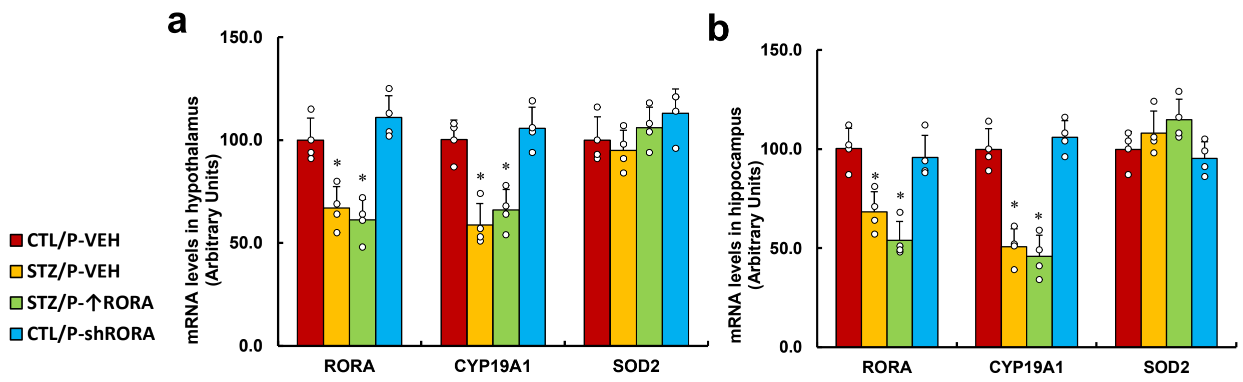

Supplementary Figure 8. Potential effect of postnatal expression of RORA on maternal diabetes-induced gene expression in offspring. At 6 weeks of age, male offspring from either the control (CTL) or maternal diabetes (STZ) groups received either vehicle (P-VEH) or lentivirus infusion for either RORA expression (P-↑RORA) or knockdown (P-shRORA). The male offspring were then sacrificed at 8 weeks of age and the tissues for hypothalamus and hippocampus were isolated for gene analysis. (a) mRNA levels in hypothalamus, n=4. (b) mRNA levels in hippocampus, n=4. *, P<0.0001, vs CTL/P-VEH group. Data were expressed as mean ± SEM.

Supplementary Figure 9

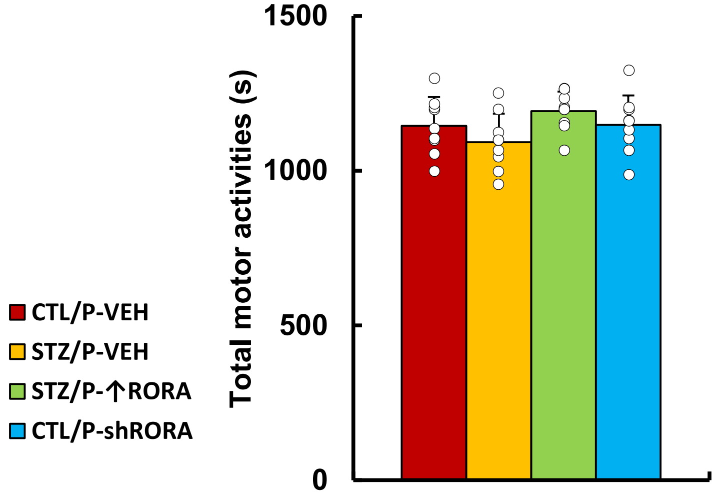

Supplementary Figure 9. Maternal diabetes and postnatal manipulation of RORA in the amygdala have no effect on total motor activity in offspring. Male offspring from either the control (CTL) or maternal diabetes (STZ) groups received either vehicle (P-VEH) or lentivirus infusion for either RORA expression (P-↑RORA) or RORA knockdown (P-shRORA) at 6 weeks old, and the subsequent 7-8 week-old male offspring were used for analysis of total motor activities, n=9. Data were expressed as mean ± SEM.

Supplementary Figure 10.

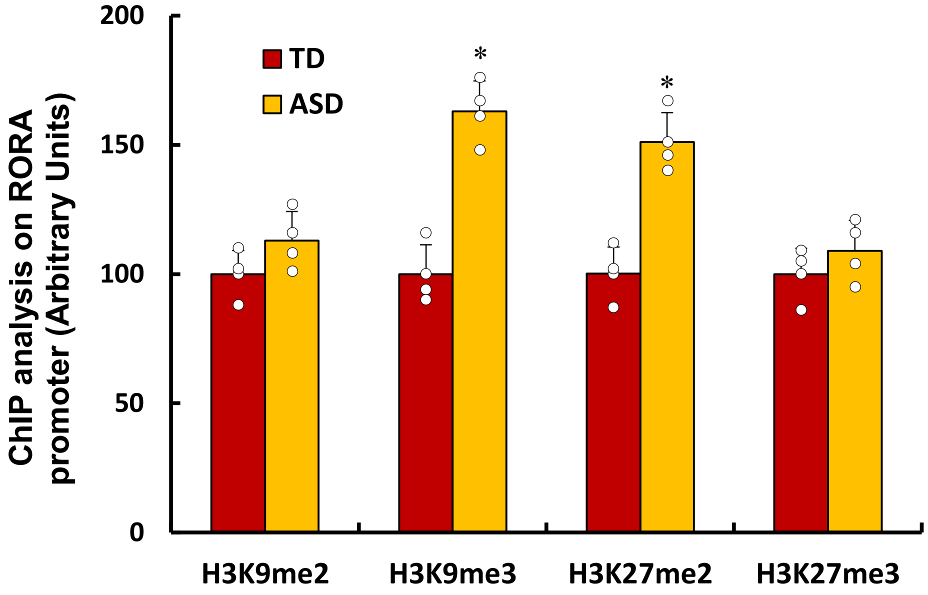

Supplementary Figure 10. Epigenetic modifications on the RORA promoter in human PBMC. 3 ml of peripheral blood was withdrawn from either typical developing (TD) and ASD children (2-6 years old). The PBMC were isolated and combined PBMC (n=30) from the TD or ASD groups, respectively, were used for ChIP analysis, n=4. *, $P<0.0001$, vs TD group. Data were expressed as mean ± SEM.

Supplementary Figure 11

a

Coordinates of the Curve

Test Result Variable(s): ASD2

Cut/Off Value → .530550

Positive if Greater Than or Equal To[a]	Sensitivity	1 - Specificity
-.858100	1.000	1.000
.210650	1.000	.992
.279700	1.000	.983
.286700	1.000	.975
.295250	1.000	.967
.304900	1.000	.959
.330800	1.000	.950
.352350	1.000	.942
.366000	1.000	.934
.382800	1.000	.926
.396550	1.000	.917
.411000	1.000	.909
.428400	1.000	.901
.444400	1.000	.893
.454200	1.000	.884
.472350	1.000	.876
.488150	1.000	.868
.496900	1.000	.860
.506150	1.000	.851
.508400	1.000	.843
.510150	1.000	.835
.512400	1.000	.826
.516100	1.000	.818
.524000	1.000	.810
.530550	1.000	.802
.532750	1.000	.785
.535400	1.000	.777
.542550	1.000	.769
.561700	1.000	.760
.583700	1.000	.752
.601200	1.000	.744
.611500	1.000	.736
.618300	1.000	.727
.634150	1.000	.719
.651500	1.000	.711
.669500	.992	.711
.683050	.992	.702
.687650	.983	.702
.694500	.983	.694
.702850	.975	.694
.710650	.975	.686
.716650	.975	.678
.720150	.975	.669
.723650	.975	.661
.725100	.975	.653
.729000	.975	.645
.737050	.966	.645

b

.737050	.966	.645
.747150	.958	.645
.755350	.958	.636
.760300	.958	.628
.765200	.958	.620
.770050	.958	.612
.773350	.958	.603
.782200	.958	.595
.790900	.958	.587
.792200	.958	.579
.793650	.958	.570
.796850	.958	.562
.800000	.958	.554
.804950	.949	.554
.810000	.949	.545
.816150	.949	.537
.827150	.941	.537
.834100	.932	.537
.837800	.932	.529
.843050	.924	.529
.863250	.924	.521
.881150	.915	.521
.882400	.907	.521
.883500	.907	.512
.884450	.907	.504
.889150	.907	.496
.903900	.907	.488
.920550	.907	.479
.927850	.907	.471
.935800	.898	.471
.948050	.890	.471
.977550	.890	.463
1.002700	.881	.463
1.016400	.873	.463
1.029650	.873	.455
1.032600	.864	.455
1.038450	.864	.446
1.045200	.864	.438
1.051400	.856	.438
1.063450	.856	.430
1.085500	.856	.421
1.099400	.856	.413
1.100850	.847	.413
1.105650	.847	.405
1.113800	.847	.397
1.126500	.839	.397
1.135500	.839	.388
1.160500	.839	.380
1.192300	.839	.372
1.201800	.831	.372
1.204650	.822	.372
1.206250	.822	.364
1.209250	.814	.364

c

1.209250	.814	.364
1.243350	.814	.355
1.275550	.805	.355
1.279650	.805	.347
1.296250	.797	.347
1.350300	.797	.339
1.396850	.797	.331
1.421450	.788	.331
1.461550	.788	.322
1.502350	.780	.322
1.530050	.771	.322
1.540900	.763	.322
1.548300	.754	.322
1.555850	.754	.314
1.560800	.754	.306
1.562400	.746	.306
1.563300	.746	.298
1.578650	.737	.298
1.602600	.729	.298
1.626850	.720	.298
1.650950	.712	.298
1.661250	.712	.289
1.676400	.712	.281
1.751750	.712	.273
1.814550	.712	.264
1.843200	.703	.264
1.871250	.703	.256
1.875650	.703	.248
1.881450	.695	.248
1.903550	.686	.248
1.962550	.686	.240
2.013100	.686	.231
2.025400	.678	.231
2.033800	.678	.223
2.042650	.669	.223
2.051650	.669	.215
2.060100	.669	.207
2.074050	.669	.198
2.088650	.661	.198
2.095750	.661	.190
2.105100	.653	.190
2.109500	.644	.190
2.111200	.644	.182
2.120450	.644	.174
2.143000	.644	.165
2.164150	.644	.157
2.184850	.644	.149
2.238950	.636	.149
2.282150	.636	.140
2.286650	.636	.132
2.293600	.627	.132
2.306750	.627	.124
2.316000	.619	.124

d

2.316000	.619	.124
2.320850	.610	.124
2.330700	.602	.124
2.341000	.593	.124
2.356550	.585	.124
2.370450	.585	.116
2.377000	.576	.116
2.395600	.576	.107
2.412250	.568	.107
2.430650	.568	.099
2.483300	.559	.099
2.532200	.559	.091
2.554950	.559	.083
2.566600	.551	.083
2.570350	.542	.083
2.575150	.542	.074
2.617550	.534	.074
2.701700	.534	.066
2.752800	.534	.058
2.763050	.525	.058
2.770400	.517	.058
2.785650	.508	.058
2.814900	.508	.050
2.838000	.500	.050
2.854650	.500	.041
2.880250	.492	.041
2.906850	.492	.033
2.955700	.492	.025
3.001050	.483	.025
3.025250	.475	.025
3.075500	.466	.025
3.113600	.466	.017
3.138250	.458	.017
3.160350	.449	.017
3.162550	.441	.017
3.174050	.441	.008
3.203400	.432	.008
3.230050	.424	.008
3.312100	.424	.000
3.400400	.415	.000
3.431300	.407	.000
3.483550	.398	.000
3.608100	.390	.000
3.722450	.381	.000
3.809650	.373	.000
3.892850	.364	.000
4.012950	.356	.000
4.153350	.347	.000
4.372550	.339	.000
4.648800	.331	.000
4.774250	.322	.000
4.848350	.314	.000

e

4.848350	.314	.000
4.935150	.305	.000
5.075450	.297	.000
5.210850	.288	.000
5.284350	.280	.000
5.334550	.271	.000
5.375150	.263	.000
5.446900	.254	.000
5.541350	.246	.000
5.634000	.237	.000
5.687700	.229	.000
5.751400	.220	.000
5.805750	.212	.000
5.840750	.203	.000
5.893150	.195	.000
5.919450	.186	.000
5.961600	.178	.000
6.022000	.169	.000
6.060450	.161	.000
6.126100	.153	.000
6.236800	.144	.000
6.333450	.136	.000
6.489100	.127	.000
6.706600	.119	.000
6.965250	.110	.000
7.217500	.102	.000
7.331050	.093	.000
7.371450	.085	.000
7.486000	.076	.000
7.711500	.068	.000
7.925200	.059	.000
8.026450	.051	.000
8.175250	.042	.000
8.458800	.034	.000
9.147550	.025	.000
9.751400	.017	.000
9.937750	.008	.000
11.055600	.000	.000

a. The smallest cutoff value is the minimum observed test value minus 1, and the largest cutoff value is the maximum observed test value plus 1. All the other cutoff values are the averages of two consecutive ordered observed test values.

Supplementary Figure 11. Establishment of Cut/Off value for the diagnosis of ASD patients based on RORA mRNA expression. (a) The coordinates of the curve, part a. (b) The coordinates of the curve, part b. (c) The coordinates of the curve, part c. (d) The coordinates of the curve, part d. (e) The coordinates of the curve, part e. The number marked by the red arrow indicates the Pass/Fail Cut/Off value.
