# Supplementary material for: Maternal diabetes-mediated RORA suppression in mice contributes to autism-like offspring through inhibition of aromatase
Source: Commun Biol. 2022 Jan 13;5:51. doi: 10.1038/s42003-022-03005-8 (PMC8758718; doi:10.1038/s42003-022-03005-8)
Supplement: Supplementary file 2 — Description of Additional Supplementary Files [file 42003_2022_3005_MOESM2_ESM.pdf]

Description of Additional Supplementary Files

File name: Supplementary Data 1
Description: In vitro cell culture study except Fig 1a and 1b.

File name: Supplementary Data 2
Description: In vitro cell culture study for Fig 1a and 1b.

File name: Supplementary Data 3
Description: In vitro cell culture study for mapping of RORA/CYP19A11 regulation elements.

File name: Supplementary Data 4
Description: In vivo mouse study.

File name: Supplementary Data 5
Description: In vivo human study.
