# Supplementary material for: Maternal diabetes-mediated RORA suppression in mice contributes to autism-like offspring through inhibition of aromatase
Source: Commun Biol. 2022 Jan 13;5:51. doi: 10.1038/s42003-022-03005-8 (PMC8758718; doi:10.1038/s42003-022-03005-8)
Supplement: Supplementary file 8 — Reporting Summary [file 42003_2022_3005_MOESM8_ESM.pdf]

nature portfolio

Corresponding author(s): PAUL YAO; COMMSBIO-21-2921-A
Last updated by author(s): Nov 1, 2021

Reporting Summary

Nature Portfolio wishes to improve the reproducibility of the work that we publish. This form provides structure for consistency and transparency in reporting. For further information on Nature Portfolio policies, see our Editorial Policies and the Editorial Policy Checklist.

Statistics

For all statistical analyses, confirm that the following items are present in the figure legend, table legend, main text, or Methods section.

n/a	Confirmed	
☐	☒	The exact sample size (n) for each experimental group/condition, given as a discrete number and unit of measurement
☐	☒	A statement on whether measurements were taken from distinct samples or whether the same sample was measured repeatedly
☐	☒	The statistical test(s) used AND whether they are one- or two-sided<br>*Only common tests should be described solely by name; describe more complex techniques in the Methods section.*
☐	☒	A description of all covariates tested
☐	☒	A description of any assumptions or corrections, such as tests of normality and adjustment for multiple comparisons
☐	☒	A full description of the statistical parameters including central tendency (e.g. means) or other basic estimates (e.g. regression coefficient) AND variation (e.g. standard deviation) or associated estimates of uncertainty (e.g. confidence intervals)
☐	☒	For null hypothesis testing, the test statistic (e.g. F, t, r) with confidence intervals, effect sizes, degrees of freedom and P value noted<br>*Give P values as exact values whenever suitable.*
☒	☐	For Bayesian analysis, information on the choice of priors and Markov chain Monte Carlo settings
☒	☐	For hierarchical and complex designs, identification of the appropriate level for tests and full reporting of outcomes
☒	☐	Estimates of effect sizes (e.g. Cohen's d, Pearson's r), indicating how they were calculated

Our web collection on statistics for biologists contains articles on many of the points above.

Software and code

Policy information about availability of computer code

Data collection	SPSS 22
Data analysis	SPSS 22

For manuscripts utilizing custom algorithms or software that are central to the research but not yet described in published literature, software must be made available to editors and reviewers. We strongly encourage code deposition in a community repository (e.g. GitHub). See the Nature Portfolio guidelines for submitting code & software for further information.

Data

Policy information about availability of data

All manuscripts must include a data availability statement. This statement should provide the following information, where applicable:
- Accession codes, unique identifiers, or web links for publicly available datasets
- A description of any restrictions on data availability
- For clinical datasets or third party data, please ensure that the statement adheres to our policy

All the data are available either in this manuscript or supplementary information

Field-specific reporting

Please select the one below that is the best fit for your research. If you are not sure, read the appropriate sections before making your selection.

☒ Life sciences ☐ Behavioural & social sciences ☐ Ecological, evolutionary & environmental sciences

For a reference copy of the document with all sections, see nature.com/documents/nr-reporting-summary-flat.pdf

Life sciences study design

All studies must disclose on these points even when the disclosure is negative.

Sample size	For in vitro cell study, sample size n=5, animal study, sample size n=9, human study, sample size n=121
Data exclusions	All the data does not meet the acceptance criteria and all the outliers are excluded
Replication	replication n=3
Randomization	Animal study are randomized
Blinding	Both animal and humans study are double-blinded

Reporting for specific materials, systems and methods

We require information from authors about some types of materials, experimental systems and methods used in many studies. Here, indicate whether each material, system or method listed is relevant to your study. If you are not sure if a list item applies to your research, read the appropriate section before selecting a response.

Materials & experimental systems

n/a	Involved in the study	
☐	☒	Antibodies
☐	☒	Eukaryotic cell lines
☒	☐	Palaeontology and archaeology
☐	☒	Animals and other organisms
☐	☒	Human research participants
☒	☐	Clinical data
☒	☐	Dual use research of concern

Methods

n/a	Involved in the study	
☒	☐	ChIP-seq
☒	☐	Flow cytometry
☒	☐	MRI-based neuroimaging

Antibodies

Antibodies used	Antibodies for β-actin (sc-47778), C/EBPα (sc-365318), CYP19 (sc-374176), NF1 (sc-74444), Oct-3/4 (sc-5279), Pit1 (sc-393943), PU.1 (sc-390405), RORA (sc-518081), RXRα (sc-515929), SOD2 (sc-30080), Sp1 (sc-17824), SRF (sc-25290) and SYP (sc-17750) were obtained from Santa Cruz Biotechnology. Antibody for 8-oxo-dG (4354-MC-050) was purchased from Novus Biologicals; NeuN (#24307) was purchased from Cell Signaling. Antibodies for acetyl-histone H4 K5, K8, K12, and K16 (H4K5,8,12,16ac, #PA5-40084) were obtained from Invitrogen. Antibodies for histone H3 acetyl K9, K14, K18, K23, K27(H3K9,14,18,23,27ac, ab47915), H4K20me1 (ab9051), H4K20me3 (ab9053), H4R3me1 (ab17339), H3K9me2 (ab1220), H3K9me3 (ab8898), H3K27me2 (ab24684) and H3K27me3 (ab6002) were obtained from Abcam
Validation	The validation can be tracked through the serial number provided above from the vendor's website.

Eukaryotic cell lines

Policy information about cell lines

Cell line source(s)	Neural Progenitor Cells (NPC) ACS-5003 (obtained from ATCC)
Authentication	obtained from ATCC
Mycoplasma contamination	not applicable
Commonly misidentified lines (See ICLAC register)	not applicable

Animals and other organisms

Policy information about studies involving animals; ARRIVE guidelines recommended for reporting animal research

Laboratory animals	The RORAfl/fl mouse, which has loxP flanking sites targeting exon 3 of the RORA gene, was generated by in vitro fertilization and was obtained for the study as a generous gift from Dr. Haimou Zhang from Hubei University. The OtpCre mouse (#030557), which expresses Cre recombinase in hypothalamic and medial amygdala neurons, was obtained from Jackson Laboratories. To generate neuron-specific RORA-/- null mice (OtpCre-RORAfl/fl), RORAfl/fl mice were cross-bred with OtpCre mice for over 4 generations on the C57BL/6J background. Positive offspring were confirmed by genotyping through PCR using specific primers (see Table S1) for the presence of both loxP sites within RORA alleles and Cre recombinase
Wild animals	not applicable
Field-collected samples	not applicable
Ethics oversight	The animal protocol conformed to US NIH guidelines (Guide for the Care and Use of Laboratory Animals, No. 85-23, revised 1996) and was reviewed and approved by the Institutional Animal Care and Use Committee from Foshan Maternity and Child Health Care Hospital.

Note that full information on the approval of the study protocol must also be provided in the manuscript.

Human research participants

Policy information about studies involving human research participants

Population characteristics	Each autistic child was rigorously matched with one control subject of the typically developing (TD) group. 121 cases of ASD children and 118 cases of matched TD children (2-6 years old) were ultimately identified after exclusion of outliers, and participants were included in this study with informed written consent from their parents.
Recruitment	Both autistic and TD children were assessed for verbal IQ (intelligence quotient), performance IQ and full-scale IQ using the Wechsler Intelligence Scale for Children IV (WISC-IV). As a result, children from the the autistic and TD groups did not differ significantly in age, sex, total IQ, performance IQ and verbal IQ. ASD diagnosis was based on several clinical assessments by a multidisciplinary team and was further confirmed by licensed clinical psychologists and psychiatrists at Hainan Women and Children's Medical Center using the DSM-5 (Diagnostic and Statisti¬cal Manual of Mental Disorders, Fifth Edition) as the diagnostic criteria
Ethics oversight	The human study was carried out in accordance with the recommendations of the Human Subject Guidelines of the Human Subjects Institutional Review Board. The protocol was approved by Hainan Women and Children's Medical Center, and all subjects gave written informed consent in accordance with the Declaration of Helsinki. The study of human subjects was approved by the Human Subjects Institutional Review Board at the Hainan Women and Children's Medical Center.All participants were included in this study with informed written consent from their parents.

Note that full information on the approval of the study protocol must also be provided in the manuscript.
